# Cost-effectiveness analysis of population-based tobacco control strategies in the prevention of cardiovascular diseases in Tanzania

**DOI:** 10.1371/journal.pone.0182113

**Published:** 2017-08-02

**Authors:** Frida Ngalesoni, George Ruhago, Mary Mayige, Tiago Cravo Oliveira, Bjarne Robberstad, Ole Frithjof Norheim, Hideki Higashi

**Affiliations:** 1 Ministry of Health, Community Development, Gender, Elderly and Children, Dar es Salaam, Tanzania; 2 Department of Global Public Health and Primary Care, University of Bergen, Bergen, Norway; 3 Muhimbili University of Health and Allied Sciences, Dar es Salaam, Tanzania; 4 Tanzania National Institute of Medical Research, Dar es Salaam, Tanzania; 5 Institute of Health Metrics and Evaluation, Seattle, Washington, United States of America; 6 Centre for International Health, University of Bergen, Bergen, Norway; 7 Japan International Cooperation Agency, Lusaka, Zambia; Central Queensland University, AUSTRALIA

## Abstract

**Background:**

Tobacco consumption contributes significantly to the global burden of disease. The prevalence of smoking is estimated to be increasing in many low-income countries, including Tanzania, especially among women and youth. Even so, the implementation of tobacco control measures has been discouraging in the country. Efforts to foster investment in tobacco control are hindered by lack of evidence on what works and at what cost.

**Aims:**

We aim to estimate the cost and cost-effectiveness of population-based tobacco control strategies in the prevention of cardiovascular diseases (CVD) in Tanzania.

**Materials and methods:**

A cost-effectiveness analysis was performed using an Excel-based Markov model, from a governmental perspective. We employed an ingredient approach and step-down methodologies in the costing exercise following a government perspective. Epidemiological data and efficacy inputs were derived from the literature. We used disability-adjusted life years (DALYs) averted as the outcome measure. A probabilistic sensitivity analysis was carried out with Ersatz to incorporate uncertainties in the model parameters.

**Results:**

Our model results showed that all five tobacco control strategies were very cost-effective since they fell below the ceiling ratio of one GDP per capita suggested by the WHO. Increase in tobacco taxes was the most cost-effective strategy, while a workplace smoking ban was the least cost-effective option, with a cost-effectiveness ratio of US$5 and US$267, respectively.

**Conclusions:**

Even though all five interventions are deemed very cost-effective in the prevention of CVD in Tanzania, more research on budget impact analysis is required to further assess the government’s ability to implement these interventions.

## Introduction

Tobacco consumption contributes significantly to the global burden of disease. This risk factor is attributable to seven out of ten leading causes of death globally, with cardiovascular diseases (CVD), particularly ischemic heart disease (IHD), and stroke taking two of the top three positions [1]. Tobacco kills up to one in every two of its over 1.1 billion users; as such, there is no other risk factor which represents such a high mortality rate [2]. The Global Burden of Disease (GBD) study has estimated that 25% and 23% of the disability-adjusted life years (DALYs) due to IHD and stroke respectively were attributable to tobacco use in 2013 [3]. Without comprehensive tobacco control policies, it is estimated that the annual death toll associated with tobacco smoking will rise to over 8 million over the next 20 years, with more than 80% of these deaths occurring in developing countries [4].

Even though many countries in sub-Saharan Africa (SSA) have no comprehensive data on trends in tobacco use [5], it is estimated that the prevalence of smoking will increase in many low-income countries (LICs), especially among women and youth, while in contrast it stabilizes or declines in most higher-income countries [6]. Only as recently as the early 2000s did some countries (including Tanzania) start collecting tobacco use data as part of the Demographic and Health Survey (DHS) [7]. Between 2005 and 2010, the prevalence of smoking has remained stable at 21% for males but increased for females from 0.5% to 1.4% according to Tanzania DHS (TDHS) reports [8, 9]. The World Health Organization’s (WHO) STEPwise approach to non-communicable disease (NCD) risk factor surveillance for Tanzania showed that smoking prevalence among males stands at 28% and among females it is 4.5% [10].

Globally, tobacco is associated with more than half a trillion dollars in economic damages annually [2]. The consequences of tobacco on the economies of SSA countries are substantial at both the individual and health-system levels. At the individual level, tobacco use can hinder economic development both directly and indirectly. The direct effect occurs when expenditure on tobacco takes priority over expenditure on food and education and indirectly when a high proportion of the income is used on the treatment of tobacco-related diseases. This is even more pronounced in LICs, where paying for health care mostly comes from out-of-pocket expenditure [11]. Productive time is also lost when a person is sick and cannot participate in production [12]. It follows, therefore, that CVD not only incurs lifelong disability but is also expensive to treat. Such expenses will continue to put more pressure on already constrained and weak health-care systems.

In recognition of the threat posed by tobacco use and exposure, the Framework Convention on Tobacco Control (FCTC) was adopted by the World Health Assembly in 2003, and entered into force in 2005. This international treaty prescribes evidence-based, cost-effective interventions for reducing the supply and demand for tobacco [13]. Supply-side measures include restrictions on sales to minors, while demand-side measures include, for instance, bans on tobacco advertisements and protection from smoke exposure. Tanzania is one of the 168 signatories that ratified the FCTC in 2004, meaning that the country is legally bound by the treaty’s provisions [14].

In 2003, Tanzania approved a law relating to tobacco control: the Tobacco Product (Regulation) Act (TPRA) [15]. This act regulates the manufacturing, labelling, distribution, sale and promotion of tobacco products and smoking areas. Implementation of the act has been discouraging, mainly due to the absence (until 2015) of accompanying regulations to guide its implementation [16]. Furthermore, there is no national control program within the Ministry of Health, Community Development, Gender, Elderly and Children (MoHCDGEC) to oversee the implementation of these measures. Efforts by its proponents are thus fragmented between non-governmental organizations like the Tanzania Tobacco Control Forum (TTCF) and the Tanzania Public Health Association, the government through MoHCDGEC and international organizations e.g. the WHO [17]. Conflicting messages from the Ministries of Agriculture and Finance have also hindered the effective implementation of tobacco control measures. Their motivation may have been influenced by the fact that tobacco producers are known to employ significant numbers of rural Tanzanians and are among the largest taxpayers in the country [17].

Regardless of these challenges to the employment and enforcement of the current act, the effective implementation of tobacco control measures will require evidence of what works and at what cost. While this has been extensively researched in many developed countries [18], such information is scarce for Tanzania. Since context-specific evidence is important in informing decision making [19], we aim to estimate the cost and cost-effectiveness of five population-based tobacco control strategies: advertisement bans, package labelling of tobacco products, smoke-free workplaces and public places, mass media campaigns and an increase in tobacco product taxes in the prevention of CVD in Tanzania.

## Methods

Two models were constructed in Microsoft Excel: prevalence and epidemiological models. The former (prevalence model) was used to estimate smoking initiation and cessation rates, and the latter (epidemiological model) combined these rates with other epidemiological parameters, along with the cost and effectiveness of the analyzed tobacco interventions to derive the cost-effectiveness ratios (CERs) among the base population.

### Prevalence model

**Fig 1:**
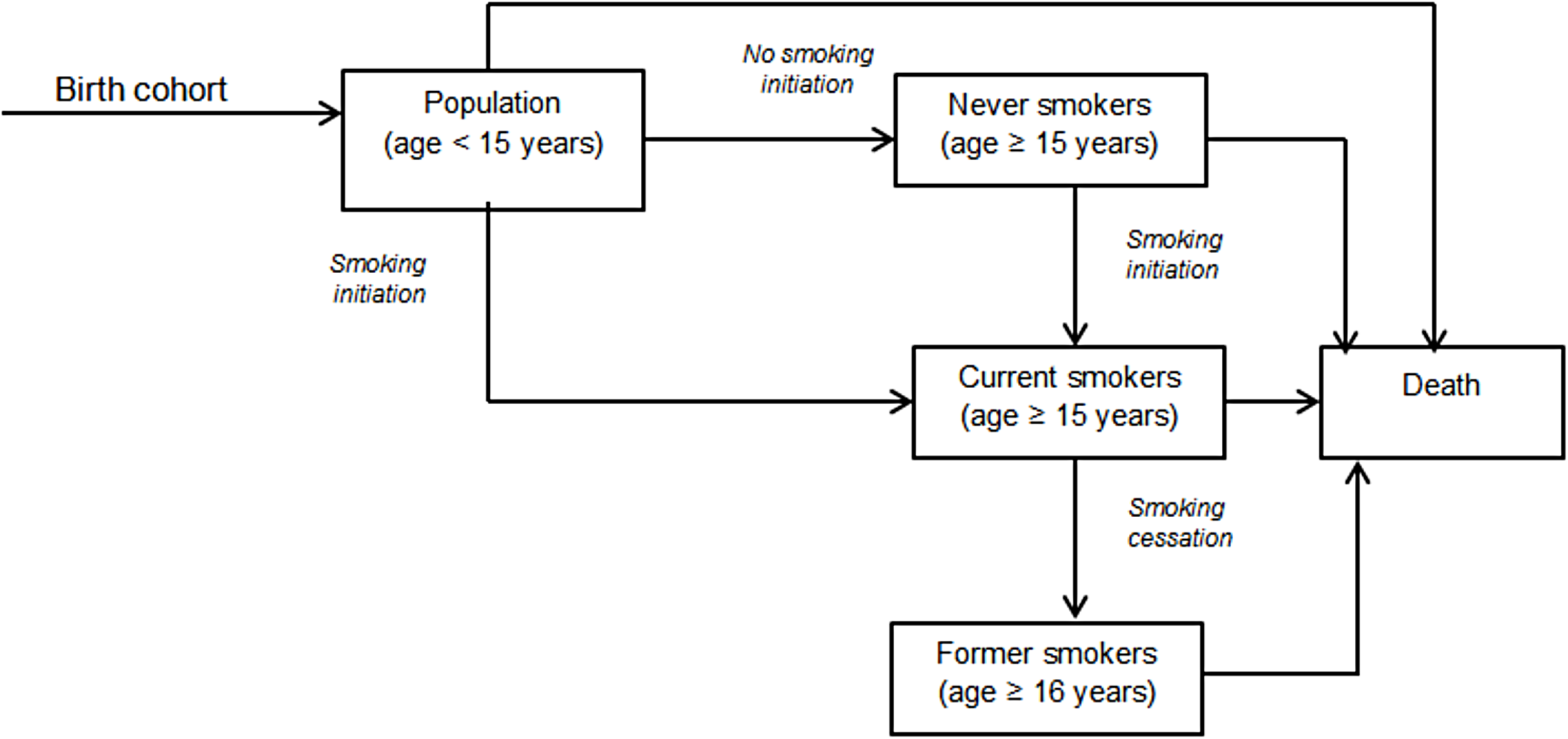
Overview of the smoking prevalence model for the base year population.

We adopted the prevalence model (see Fig 1) from original developers Mendez et al. [20] and contextualized it with data from Tanzania to estimate smoking initiation and cessation rates. The model has been used in similar studies in Australia, Italy and Vietnam [21–23].

#### Data

Observed smoking prevalence

The observed past prevalence among never smokers, current smokers (daily cigarette/tobacco smokers) and former smokers for the age groups 25–34, … 65–74 and ≥ 75 were based on survey data for 2002 and 2012 [10, 24]. For the age groups < 25 we used data from the Global Youth Tobacco Survey (GYTS) carried out in 2003 and 2008 among adolescents [25], which was assumed to reflect the smoking pattern of this age group. Data from the recent Tanzania DHS, though nationally representative, was not chosen as one of the data sources for past prevalence because data were aggregated in different age groupings (which did not match the other two data sources), the definition of current smokers was not clear and the category of former smokers was not reported. These smoking prevalence figures were then interpolated and extrapolated linearly to obtain data for the missing years. Table 1 presents these values from the data sources mentioned above.

**Table 1:** Observed smoking prevalence in Tanzania.

	Current		Former		Never		
Age	Males	Females	Males	Females	Males	Females	Source
**2002**							
<25	0.108	0.069	0.157	0.077	0.735	0.854	
25–34	0.235	0.008	0.078	0.012	0.687	0.980	
35–44	0.240	0.010	0.109	0.028	0.651	0.962	
45–54	0.211	0.028	0.144	0.037	0.644	0.935	[24, 25]
55–64	0.197	0.053	0.186	0.081	0.616	0.866	
65–74	0.174	0.042	0.229	0.089	0.597	0.869	
>75	0.224	0.039	0.265	0.072	0.512	0.889	
**2012**							
<25	0.091	0.065	0.134	0.088	0.775	0.847	
25–34	0.169	0.004	0.317	0.021	0.514	0.976	
35–44	0.251	0.008	0.387	0.038	0.362	0.955	
45–54	0.292	0.068	0.490	0.089	0.218	0.843	[10, 25]
55–64	0.236	0.041	0.476	0.131	0.288	0.828	
65–74	0.263	0.039	0.457	0.091	0.280	0.870	
>75	0.247	0.039	0.422	0.096	0.331	0.865	

Calculated smoking prevalence

Probability of dying

The annual probabilities of dying in never, current and former smokers were estimated from the age and sex-specific mortality rates for each smoking status. These mortality rates were calculated from i) all-cause population mortality rates based on a Tanzanian life table for 2013 [26], ii) the observed smoking prevalence from the data sources explained above and iii) the relative risk of mortality according to smoking status from the Cancer Prevention Study (CPS) phase II (M Thun, personal communication). The use of a US-based study was motivated by the lack within Tanzania of any such large-scale, context-specific prospective studies which followed up millions of individuals with different smoking status over a long period. The following formulas were applied to estimate the mortality rates and probabilities of dying [22]:

$$M_{x,t}^{n}=\frac{M_{x,t}^{p}}{{RR}_{x}^{c}\;x\;{Prev}_{x,t}^{c}+{RR}_{x}^{f}\;x\;{Prev}_{x,t}^{f}+1\;x\;1-({Prev}_{x,t}^{c}+{Prev}_{x,t}^{f})}$$

$$M_{x,t}^{c}={RR}_{x}^{c}\;x\;M_{x,t}^{n}$$

$$M_{x,t}^{f}={RR}_{x}^{f}\;x\;M_{x,t}^{n}$$

Where,

$M_{x,t}^{n},\;M_{x,t}^{c},\;M_{x,t}^{f}$ = mortality rate for never, current and former smoking status respectively in age group *x* and year *t*

$M_{x,t}^{p}$ = mortality rate of the total population in age group *x* and year *t*

${Prev}_{x,t}^{c},\;{Prev}_{x,t}^{f}$ = prevalence of current and former smokers respectively in age group *x* and year *t*

${RR}_{x,t}^{c},\;{RR}_{x,t}^{f}$ = relative risk of mortality in current and former smokers respectively compared to never smokers in age group *x* and year *t*. The mortality rates (MR) for never, current and former smokers for age group *x* and year *t* were then converted into the probability of dying for age group *x* and year *t* using the formula: *P* = 1 − *e*^−*MR*^ [27]

Number of smokers

We inferred the population size for the base year 2013 from the 2012 census by applying age-specific fertility rates, sex ratio and mortality rates [28]. For the years analyzed, the population was divided into never, current and former smokers based on: i) probability of dying according to smoking status, ii) the proportional initiation and cessation among never and current smokers respectively, and iii) fertility and sex ratio obtained and extrapolated from the Tanzanian censuses of 2002 and 2012 and TDHS 2010 [9, 28, 29]. The formulas used to obtain these numbers are presented in S1 Text. The estimated smoking prevalence was calculated as the ratio of the number of individuals according to smoking status in a particular age group to the total number of individuals in that age group.

Initiation and cessation rates

To estimate the unknown age-specific initiation and cessation rates in the model, a set of values for these parameters that best fits the observed smoking prevalence was estimated by means of an optimization routine. We used the generalized reduced gradient algorithm optimization technique embedded in Solver, which is a Microsoft Excel add-in function. During this process, we employed the weighted least squares method [20–23] using the inverse of the variance of the observation as a set of weights for the observed prevalence to reflect the fact that the different age groups in the four surveys have different sample sizes [30]. The estimated initiation and cessation rates were assumed to be constant throughout the cohort’s lives unless they were subjected to intervention effects (Table 2).

**Table 2:** Smoking initiation and cessation rates for the base year 2013.

Age	Males		Females	
	Mean	Std. deviation	Mean	Std. deviation
<25	-0.0147	0.0076	-0.1801	0.0089
25–34	-0.0144	0.0024	0.5088	0.0441
35–44	0.0004	0.0079	0.0199	0.0242
45–54	-0.0177	0.0060	-0.0032	0.0010
55–64	-0.0039	0.0109	0.1200	0.0493
65–74	-0.0316	0.0108	0.0421	0.0441
>75	-0.0356	0.0110	0.0333	0.0474

Minus signs indicate smoking initiation otherwise the value indicates smoking cessation

Representing uncertainty in initiation and cessation rates

Uncertainty intervals for these initiation and cessation rates were estimated by Monte Carlo simulation with 10,000 iterations using Ersatz bootstrap software [31] by resampling the input parameters from their assumed distributions. For relative risks of mortality according to smoking status, lognormal distributions were fitted, while Dirichlet distributions were used for the prevalence of never, current and former smokers. A beta distribution was used for smoking cessation and initiation rates. These distributions were modified to have a lower bound of -1 to allow for negative smoking initiation rates (i.e. cessation).

### Epidemiological model

#### Decision model

A Markov model previously published in a similar work in Vietnam [22] was used to bring together smoking initiation and cessation rates from the prevalence model, epidemiological parameters, and the intervention cost and effect in order to estimate the costs, health outcomes and cost-effectiveness ratios of five tobacco control strategies from a Tanzanian government perspective.

**Fig 2:**
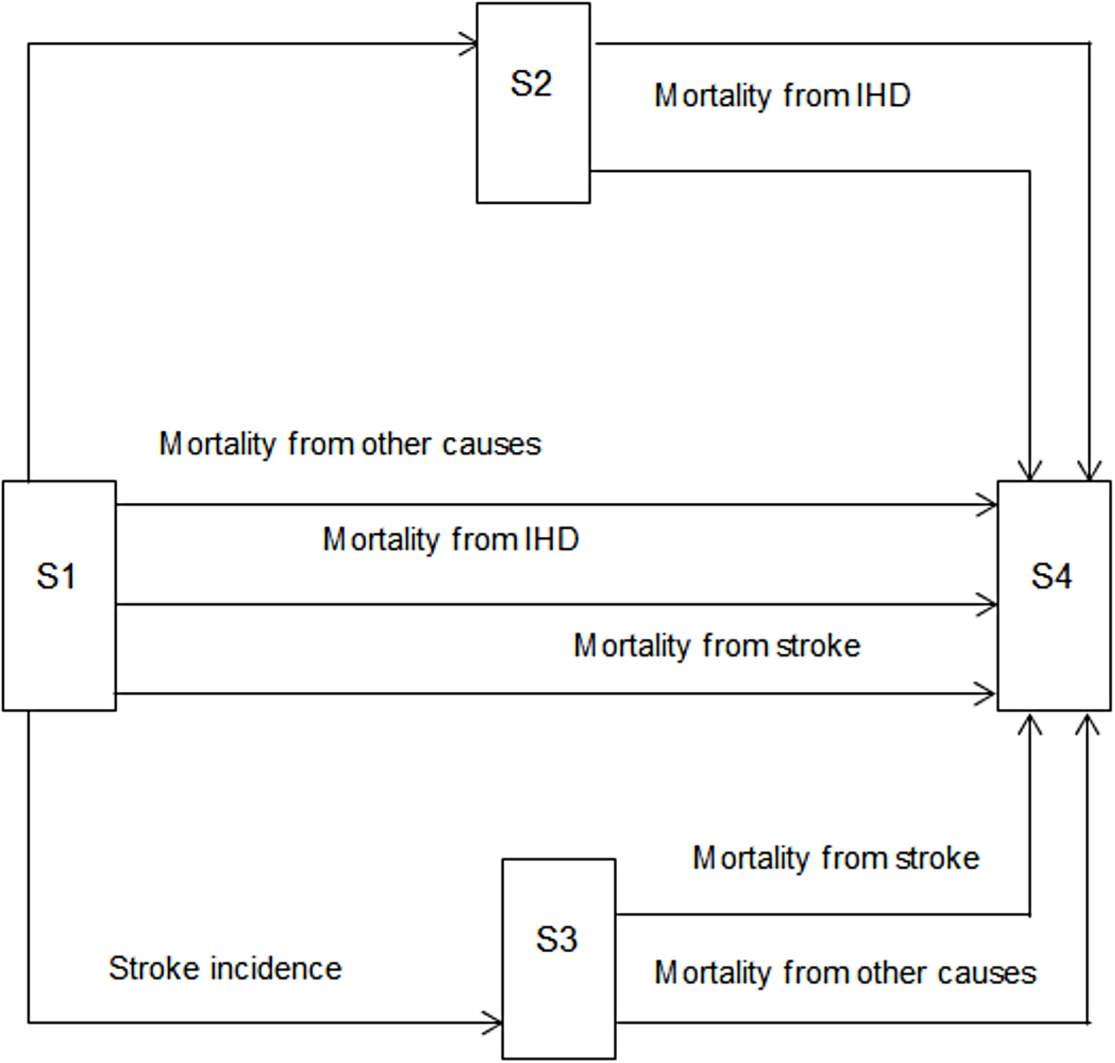
Overview of the epidemiological Markov model. Health states: S1 = No history of CVD (i.e. IHD or stroke), S2 = Histroy of IHD, S3 = History of stroke and S4 = Dead.

Four mutually exclusive health states were considered: “no history of CVD” (i.e. no previous IHD or stroke), “history of IHD”, and “history of stroke”. Finally, “death” was modelled as an absorbing state (Fig 2). The Markov cohort consisted of 5-year age-groups of estimated Tanzanian population from 15–80+ years of age, i.e. 15–19, 20–24… 80+. The first cohort of 15–19 years was followed up until they were 80+. Since we assumed that smoking initiation does not take place before 15 years of age, this cohort enters the model from the “no history of CVD” health state and transits between the different health states according to age-specific risks for each type of clinical event and depending on their risk profiles being influenced by their smoking status (with and without adjustments from the intervention effects). The second to the fourteenth cohort basically follow the same structure; however, depending on the estimated incidence values of the two diseases modelled that mirror the other epidemiological parameters for that age-group, some cohorts did not always start at the “no history of CVD” health state.

In the model, the risk of IHD and stroke were adjusted according to smoking status by calculating the Potential Impact Factor, using the formula below [32]:

PIF=∑x=12PrevxxRRx−∑x=22Prevx^xRRx∑x=22PrevxxRRx

Where:

*Prev*_*x*_ is the population smoking prevalence distribution for the cohort in base year 2013, Prevx^ is the future smoking prevalence before and after interventions, and RR is the relative risk of contracting a disease depending on smoking status (Table 3), with 1 standing for non-smokers and 2 for smokers.

**Table 3:** Annual risk of IHD and stroke among smokers compared to non-smokers.

Disease	Age	Male			Female		
		RR	95% LCI	95% HCI	RR	95% LCI	95% HCI
IHD	35–64	2.6	2.4	2.9	3.2	2.8	3.6
	>65	1.5	1.3	1.6	1.7	1.6	1.9
Stroke	35–64	2.4	1.8	3.0	3.8	3.1	4.7
	>65	1.5	1.2	1.8	1.6	1.4	1.9

IHD = Ischemic heart disease, RR = Relative risk, LCI = Low confidence interval, HCI = High confidence interval.

Source [33].

At the end of each annual cycle, the health outcomes and costs associated with the different health states were evaluated and accumulated.

#### Description of interventions

Five interventions namely advertisement, promotion and sponsorship bans, package labelling of tobacco products, smoke-free public places, mass media campaigns, and increasing the taxation on tobacco products were modelled. Table 4 provides further descriptions of the interventions, see S2 Text for a detailed decription). The scope of the measures included initial investment in revising the legislation, promotion and advocacy in the first year, further sensitization and training in the third year and five years of ongoing management and law-enforcement activities.

**Table 4:** Description of the tobacco control interventions analysed.

Intervention	In country regulatory status	WHO FCTC compliant/alignment status	Assumptions for analysis	Source
Advertisement, promotion and sponsorship ban.	No comprehensive ban. Few forms of tobacco advertisment and promotion are prohibited specifically in radio and television but it is unclear if the ban applies to domestic print. There are some restrictions on tobacco sponsorship and the publicity of such sponsorship.	To align with FCTC guidelines, the law should prohibit all tobacco advertising and promotion, including in domestic newspapers and magazine. To clarify the scope of the ban, the law should provide a definition of “tobacco advertising and promotion” in accordance with the definition provided in FCTC.	Comprehensive ban on advertisement in all media outlets and ban in all promotion and sponsorship activities.	[13, 15–17, 34, 35]
Package labelling of tobacco products.	TPRA indicates that “one of ten authorized text messages” are to be displayed. There is no guidance on graphic display, size, format or placement of the health warning.	To align to FCTC, TPRA and its associated regulations should specify size, placement, format and rotation of the health messages. The message should occupy 30% - 50% of the pack and needs to be updated regularly.	Both graphic and text messages modelled with 30% face coverage.	[13, 15, 16, 34, 35]
Smoke-free public places.	Even though a public place is defined in the TPRA, public transport is not. Smoking is baned in public places, however designated smoking areas are still allowable in indoor public places.	The TPRA and its associated regulations needs to prohibit smoking in all indoor public areas including hotel rooms, prisons, public transport and workplaces.	Different scenarios pertaining cost of non-smoking signs were analyzed.	[13, 15–17, 34]
Mass media campaigns.	TPRA is silent.	The law should stipulate the relevance of information, education, communication and other mass media campaigns in the reduction of tobacco consumption.	Implementation of a number of mass media campaigns was considered. Development and promotional of educational materials was analysed.	[13, 15]
Increase in tobacco excise taxes.	The Tobacco (Imposition of Tax) Act guides the imposition of tax on tobacco sales. The total taxes on sold brand is 35% and excise taxes is less than 20%.	FTCT requires countries to adopt or maintain measures which may include implementing tax and price policies on tobacco products so as to contribute to the health objectives aimed at reducing tobacco Consumption.	Two scenario analyzed i) from current rate to 50% and ii) from current rate to maximum proposed by WHO.	[13, 17, 36]

WHO = World Health Organisation, FCTC = Framework Convention on Tobacco Control, TPRA = Tobacco Product (Regulation) Act

#### Input parameters

Transition probabilities

Data for the incidence of IHD and stroke and disease-specific mortality rates were obtained from the GBD 2013 study [37, 38]. These were further modelled using DisMod II software [39]. We assume that the incidence of each disease is independent of the other and independent of all other causes of death except its own disease-specific mortality. We also assume that all causes of death are independent of each other. Age-specific background mortality rates were based on a Tanzanian life table [26] and were adjusted for the mortality attributable to IHD and stroke.

See Table 5 for a summary of parameters and sources.

**Table 5:** Model parameters and data sources.

Parameter	Sources	Reference
**Prevalence Model**		
Population	Census 2012	[28]
Age-specific fertility rate	TDHS 2010	[9]
Sex ratio at birth	TDHS 2010	[9]
Age and sex-specific overall mortality rates	Tanzania life tables	[26]
Mortality rates for never, current and former smokers	CPSII	M Thun, personal communication
Smoking prevalence rates	Specific studies	Table 1
**Epidemiological model**		
Population	Census 2012	[28]
Incidence and prevalence rates for IHD and stroke	GBD 2013 and DisMod modelling	[37]
Disease-specific mortality rates for IHD and stroke	GBD 2013 and DisMod modelling	[38]
Age and sex-specific background mortality rates	Tanzania life tables	[26]
Age-specific disability weights	GBD 2013	[37]
RR of IHD and stroke among smokers compared to non-smokers	Specific studies	Table 3
**Others**		
Intervention effects	Specific studies	Table 6
Intervention costs	Primary data	Table 7, S1 Table

Intervention costs

**Fig 3:**
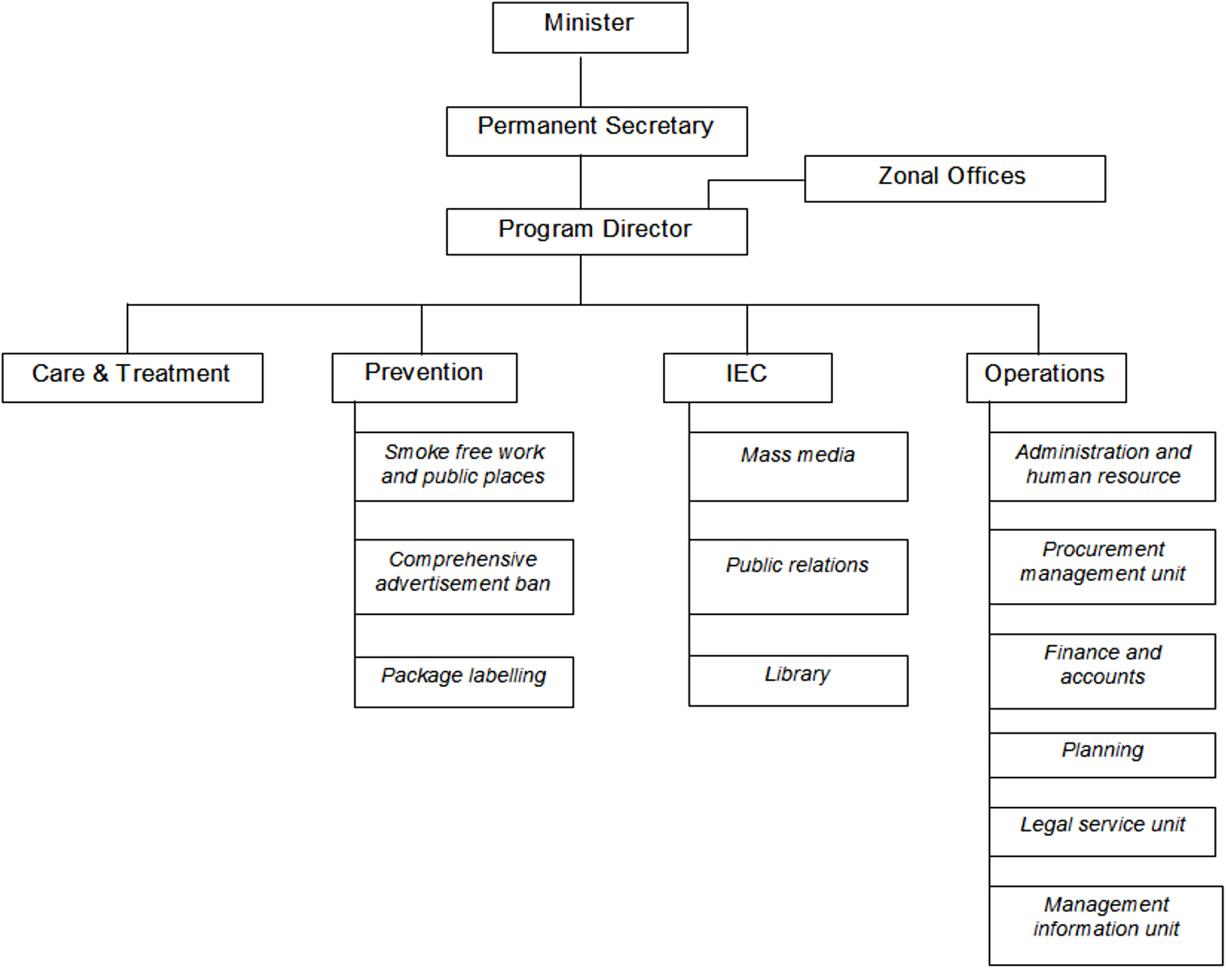
The assumed tobacco control program organogram.

Tanzania does not have an established national tobacco control program and we therefore conducted a costing study assuming an institutional setup adapted from the Tanzania Food and Drug Authority (TFDA)–with some modifications (Fig 3). Most of the primary data were collected from TFDA, which is a government organization under the MoHCDGEC responsible for regulating the quality and safety of food, drugs, cosmetics and medical devices. It should be noted that TFDA has a somewhat different mandate, however. Being a governmental organization that has both headquarters and zonal offices and is primarily concerned with regulation and law enforcement, it formed a suitable alternative for possible resource use for a potential national tobacco control program. Three (out of five) of its directorates: the office of the director general, the directorate of food safety and the directorate of business support, related well to all the interventions considered in this work except tax increases. For instance, activities under the inspection of banned/expired food products from stores and supermarkets matches well with the inspection of smoke-free areas in workplaces and public spaces. Some of the data for the human resource requirements of law enforcement were obtained from Ilala municipality, Dar es Salaam, since TFDA has a mandate to use supplementary government employees for law enforcement exercises. Cost data for the tobacco tax increase was obtained from the Tanzania Revenue Authority (TRA), which assesses, collects and accounts for all central government revenue. The departments costed included the office of the commissioner general, the Large Taxpayers’ Department (LTD), where tobacco tax revenue is dealt with, and five out of seven support departments (finance, human resource and development, information and communication technology, research and policy departments).

We conducted detailed interviews with key personnel at both institutions to determine the possible resource use for each of the interventions analyzed. We also inspected order books, inventory records, issue vouchers and delivery notes so as to record all the equipment and supplies consumed. Finance and procurement sections were consulted to determine the resources used at the administrative level and overhead costs. Building costs were obtained by measuring the area of the involved offices in square meters and, wherever necessary, physical counting of equipment was performed.

The costing period was the fiscal year from July 2011 to June 2012. The costing exercise was guided by the WHO’s tobacco costing tool, which is one of the modules in the overall non-communicable disease (NCD) costing tool [40–42]. Cost valuation followed an opportunity-cost approach, whereby all resources are valued based on their best alternative use [43]. We used the Tanzania Government Procurement Services Agency tender prices for equipment and supplies. Rental charges for buildings were calculated according to National Housing Corporation (NHC) rates. The prevailing market price was used as proxy for items whose prices were unavailable from the data sources mentioned above. All costs were estimated in Tanzanian shillings (Tshs) and converted to base year (2013) figures using a Tanzanian GDP deflator [44]. These were then converted to US$ using the mean exchange rate for 2013 of Tshs1605/US$1 [45].

A cost analysis was performed within the epidemiological model described above. For all the five interventions analyzed, we divided the resource use into six cost centers: a) strategy development and evaluation, b) human resource requirements, c) promotion, media and advocacy, d) program supplies, e) rent, equipment and office supplies and f) operations. Costs were then identified as recurrent or capital costs. Capital costs were annuitized using a rate of 9.6%, which was the average interest rate for the year 2012/13 and their useful life years were based on WHO assumptions [46, 47]. Capital items costing less than US$62 (TSh100 000) were treated as recurrent costs. A step-down costing methodology was used to allocate shared costs between the interventions using different allocation keys [48] (see S1 Table for more details on cost breakdown and their associated cost-sharing assumptions).

Intervention effects

Intervention effects were based on data from published single studies. A recent systematic review of tobacco control measures outlined the evidence for various effects estimates without pooling due to the high heterogeneity in the characteristics of interventions, level of policy enforcement and underlying tobacco control environment [49]. Most of the studies selected reported effects sizes as odds ratios (OR), in which case a 2 by 2 table was constructed from the information given and relative risk (RR) was back-calculated. With the exception of price elasticities, data was lacking from SSA or other developing countries. We therefore had to rely on data from other regions. A 10-year time horizon was assumed for intervention effects (see Table 6 for an overview of the studies considered), after which we assumed no further effects (S3 Text provides further elaboration on the quantification of intervention effects on smoking prevalence).

**Table 6:** Effect size for tobacco control interventions on smoking initiation, cessation and prevalence.

Intervention	Effect on	Country	Study design	Intervention, *n*	Smoking measure	Effect size (95% CI)	Distribution	Source
Advertisement, promotion and sponsorship ban	Prevalence	NA	Review and modelling	Various	NA	4% +/- 20%	Pert	[50]
	Initiation	NA			NA	RR = 0.94 +/- 20%	Pert	[50]
	Cessation	NA	Systematic review	No data	NA	RR* = 1	NA	[49]
Packaging labelling of tobacco products	Initiation			NR	NR	RR = 0.67 (0.49 to 0.87)	Lognormal	[51]
	Cessation	Canada	Before and after survey	Before introduction of comprehensive warning labels; after introduction of comprehensive warning labels, n = 191	Quit smoking before and after comprehensive warning labels	RR = 1.99 (1.29 to 3.05)	Lognormal	[52]
Smoke-free (public places)	Initiation	England	Longitudinal	(I) Complete ban in restaurants and/or bars, n = 632 (m) and 1,072 (f); (B) No smoking bans, n = 2,624 (m) and 4,158 (f).	Daily smoking	RR(m) = 0.83 (0.57 to 1.22); RR(f) = 0.86 (0.59 to 1.26)	Lognormal	[53]
	Cessation	UK	Longitudinal	(I) Complete ban in restaurants and/or bars in Scotland, n = 507; (B) other parts of UK, n = 828	Smoked at least once/month and at least 100 cigarettes in a lifetime	RR = 1.09 (0.91 to 1.30)	Lognormal	[54]
Smoke-free (workplaces)	Initiation	S: 1 and S: 2 USA	S:1 Prospective cohort; S:2 Longitudinal	S: 1 –(I) Smoke-free hospitals, n = 1033; (B) Non-smoke-free workplaces, n = 816. S:2 –(I) Smoke-free work area; (B) Non smoke-free work area, n = 1844	Post-ban relapse rate	RR = 1	Lognormal	[55, 56]
	Cessation	USA	Prospective cohort	(I) Smoke-free hospitals, n = 1033; (B) Non smoke-free workplace, n = 816.	Post-ban quit ratio	RR = 2.29 (1.56 to 3.37)	Lognormal	[56]
Mass media campaign	Initiation	USA	Longitudinal	(I) TV campaign with cumulative exposure between 2000–2004, n = 8,904	Ever smoked a cigarette	HR° = 0.8 (0.71 to 0.91)	Lognormal	[57]
	Cessation	USA	Longitudinal	(I) TV campaign above 1218 GRPs between 1999–2000; (B) TV campaign below 1218 GRPs between 1999–2000	NR	RR^ = 1.1 (0.98 to 1.24)	Lognormal	[58]
Increase in tobacco taxes*	Prevalence	Tanzania	Household survey	Changes in real prices 2013	NR	Ela = -0.88 (-0.78 to -0.37)	Lognormal	[12]
	Initiation	Vietnam				Ela = -1.175 +/- 20%	Pert	[59, 60]
	Cessation					Ela* = 0	NA	

Note: Studies to be included in the modelling exercise were mostly chosen from the recent systematic review by Wilson LM et al. [49], evidence differed considerably and no pooling of effects was undertaken, choice of individual studies depended on the quality reported in this review and, in a few cases, authors’ choice. n–number; CI–confidence interval; NA–not applicable; RR–relative risk; NR–not reported; S1 –Study 1; S2 –Study 2; I–intervention; TO–text only; G–graphic; B–baseline; m–males; f–females; GRPs–gross rating points

HR° –hazard ratio (effect size assumed to be the same as RR); Ela–elasticity

RR*/Ela*–assumed

RR^–reported from the primary study (all other RR estimates are calculated from OR).

#### Health outcomes

Smoking influences the transition probabilities, and in the model smoking interventions therefore translate into changes in CVD incidence and mortality which are then converted into generic health outcomes measured in disability-adjusted life years (DALYs) by using disability weights from GBD 2013. DALYs were then discounted at a rate of 3% [61]. For more details, refer to S4 Text.

#### Cost-effectiveness modelling

The expected costs and outcomes of the interventions modelled were calculated independent of each other. Base-case results are presented as average costs and effects and average cost effectiveness ratios (ICERs). Strategies having ACERs below US$910, which was Tanzania’s 2013 GDP per capita [44], (the lowest willingness to pay (WTP) value recommended by the WHO [62]) were considered “very cost-effective”.

#### Representing uncertainty

Probabilistic sensitivity analyses were performed to account for the overall model uncertainty by running all the base-case parameters concurrently as distributions using Monte Carlo simulations. We performed 2000 iterations for each of the 14 age groups modelled using Ersatz software [31]. We used PERT and a lognormal distribution for intervention costs and effects, respectively [63].

### Ethical statement

Ethical clearance was provided by the Ethical Review Committee of the Tanzania National Institute of Medical Research with Ref. No. NIMR/HQ/R.8a/Vol. IX/136. Additionally, we sought further permission to conduct the study from the Director General of Tanzania Food and Drug Authority and Commissioner General of Tanzania Revenue Authority. We were granted permission in writing and instructed to work with personnel in the relevant departments who further provided verbal informed consent to participate in the study.

## Results

The annual cost and cost-effectiveness for the five interventions analyzed are provided in Tables 7 and 8.

**Table 7:** Intervention cost of five demand-side tobacco control measures in US$.

Cost center	Advertisement, promotion and sponsorship ban	Package labelling of tobacco products	Smoke-free public spaces and workplaces	Mass media campaigns	Tobacco tax increases
Program development strategies	52,710	52,710	52,710	-	145,535
Human resource requirements	1,545,666	1,513,731	2,345,066	330,694	1,464,615
Promotion, media and advocacy	107,267	107,267	107,267	739,172	7,765
Program supplies	-	-	-	89,115	-
Rent, equipment and office supplies	30,131	28,783	30,131	71,133	21,860
Operations	885,042	885,042	885,042	885,042	27,170
**Total**	**2,620,816**	**2,587,533**	**3,420,216**	**2,115,156**	**1,666,945**

**Table 8:** Cost, effectiveness and cost-effectiveness for base-case tobacco control strategies in Tanzania.

Intervention	Cost	DALYs averted			ACER	ICER
		Males	Females	Total		
No intervention	0	0	0	0	0	_
Tobacco tax increase	1,547,355	249,126	38,706	287,832	5	5
Mass media campaigns	1,996,026	33,018	19,664	52,682	38	Dominated
Package labelling	2,248,370	44,903	11,269	56,174	40	Dominated
Advertisement ban	2,164,048	19,894	2,438	22,332	97	Dominated
Smoke-free public places	3,646,117	31,021	4,294	35,315	103	Dominated
Smoke-free workplaces	3,381,652	5,681	6,985	12,666	267	Dominated

DALYs = Disability-adjusted life years, ACER = Avergae cost-effectiveness ratio, ICER = Incremental cost-effectiveness ratio

### Costs

The total cost of the tobacco tax increase intervention was the lowest of the five interventions modelled (US$1.7 million). Human resource requirements consumed the highest proportion of total costs for all the interventions except mass media campaigns (Table 7).

### Health effects

The health impact of the assessed interventions varied dramatically, ranging from close to 2,500 to about 250,000 DALYs averted. More DALYs are averted among males than females, with the largest number of DALYs averted by tax increases (Table 8).

### Cost-effectiveness

Cost-effectiveness ratios for all five interventions were compared against the status quo scenario of no intervention for the base year. As shown in Table 8, average cost-effectiveness ratios (ACER) were found to be below US$910–which is Tanzania’s GDP per capita for 2013–per DALY averted. Interventions with a cost-effectiveness ratio below one and between one and three times the GDP per capita per health effect have been recommended by the WHO to be considered very cost-effective and cost-effective, respectively [62].

The most cost-effective strategy was a tax increase while the least cost-effective was a smoking ban in workplaces, with ACER of US$5 and US$267 per DALY averted, respectively. When incremental cost-effectiveness ratios were calculated, no intervention and increases in tobacco tax dominated all of the four remaining interventions.

### Representing uncertainty

**Fig 4:**
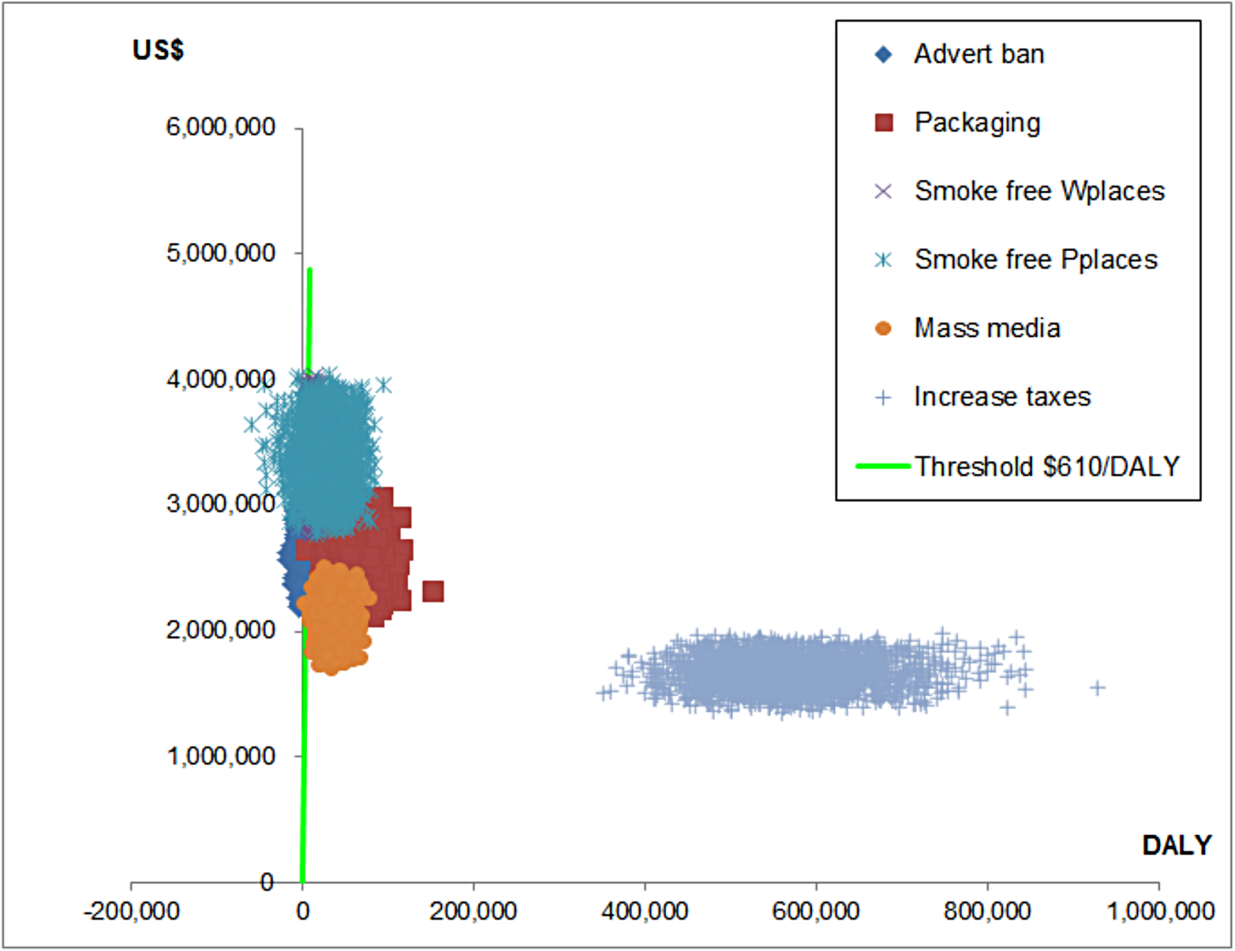
Cost effectiveness scatter plot for the tobacco control strategies. Wplaces = work places, Pplaces = public places.

Fig 4 presents a cost-effectiveness scatter plot to represent uncertainty around the model recommendations. All interventions are uncertain both in costs and effects, but the tax increase is relatively more uncertain regarding effectiveness than costs.

## Discussion

In this study we have quantified the health effects, costs, and cost-effectiveness of five demand-side tobacco control preventive strategies for reducing the risk of CVD at the population level. Our results suggest that the modelled interventions are all very cost-effective as they fall below one times the GDP per capita for Tanzania for 2013, with a tobacco tax increase yielding the most favorable cost-effectiveness ratio.

While historically much of the cost-effectiveness evidence for tobacco control interventions has come from high-income countries, data gathered and analyzed from low- and middle-income countries show similar success. Results from many studies in South Africa have consistently shown that an increase in tobacco tax is the single most cost-effective tobacco control measure [64]. Taxation has been shown to have a three-fold effect on consumption: taxes provide a barrier to initiation, reduce consumption among current smokers, and prevent former smokers from relapsing [65]. Since we did not find any studies from other context-specific settings, we also compared our model conclusions with other studies conducted in WHO and World Bank regions, and these also conform to our results [2, 5, 42, 66, 67]. A study from the Republic of Moldova reveals that tobacco taxation dominates all other demand-side individual and population-based interventions, except when all of the interventions were combined [68].

Although we did not explicitly model changes in tobacco revenue with increase in tobacco taxes our intuition point towards a “win-win” situation of increase in revenue and decrease in smoking prevalence. This is due to the opposite effect of price elasticity on prevalence and smoking initiation which when coupled with rapid Tanzanian population growth would likely lead to revenue increase [69]. Revenues generated from tobacco taxes can be substantial as shown by Goodchild et al. who estimated an increase of 85% in the tax revenue base for Africa [70]. Therefore, apart from the benefits of reducing tobacco consumption, utilizing a portion of the tobacco tax revenues to fund, for instance, mass media public awareness campaigns and cessation programs can further reduce tobacco consumption [17]. This has been achieved in many countries globally, with a notable success story reported in Thailand [71].

Health care costs associated with tobacco and tobacco-related disease expenditure have been shown to be significant [72]. Consequently, reduction in tobacco use would be expected to lower these costs. However, a few studies that have estimated the economic impact of smoking cessation in a lifetime perspective found other results [73]. Barendregt et al for instance showed that nonsmokers (never smokers and ex-smokers) have 15% higher direct lifetime health care costs due to longer life expetancies than smokers [74]. Tobacco-related illnesses and premature mortality also impose high productivity costs to the economy because of sick workers and those who die prematurely during their working years [75]. Consideration of productivity losses into economic analyses still attracts a lot of debate and inclusion or exclusion of these costs can influence results [76].

The health benefits and economic impact of tobacco control are broad and may extend far beyond the health sector. The assessment of costs and outcomes in this analysis has been limited to benefits within the health sector, following the perspective and methodology of the cost-effectiveness analysis employed. Other non-health benefits that might be important, such as productivity gains, could be explored from a broader societal perspective [77].

our modelled interventions are estimated to be very cost-effective, however, it is worth mentioning that these results should serve as inputs to the decision-making process regarding population health improvement. In LICs like Tanzania, there may still be highly cost-effective programs yet to be implemented both within and outside the health sector that compete for the same resource base.

### Strengths and limitations

Our use of initiation and cessation instead of prevalence rates alone has several advantages. Firstly, it distinguishes between never and former smokers, who have different mortality rates compared to current smokers, which cannot be captured using only prevalence rates. Secondly, modelling it this way also enabled us to capture different smoking behaviors between age groups, since the young probably demonstrate high initiation while the older have high cessation rates. Thirdly, different interventions for tobacco control act differently upon initiation and cessation rates. Lastly, our projected base-case smoking prevalence compares favourably with that estimated for Tanzania in the Global trends and projections for tobacco use (e.g. 0.198 versus 0.222 for males aged 25–64 in the year 2025) [78]. This comparison, however, should be interpreted with caution due to the different modelling approaches employed.

These results should also be considered in light of model limitations. Firstly, our adaption of this previously published model to Tanzania was not without data availability challenges. We made use of much local data on costs and some epidemiological data, but no national data were available on other epidemiological parameters; for instance: incidence, risk of death from smoking, excess mortality from tobacco-related diseases and the effectiveness of tobacco control interventions. In the absence of such information, we therefore derived epidemiological and effect estimates from other international studies and the recent GBD study. Secondly, the absence of a body that oversees tobacco control in Tanzania required us to make the conservative assumption that tobacco control can be achieved using a similar government organization, which we then used as a model for the costing exercise. Such an attempt could likely over- or under-estimate our cost estimates. We made efforts to cost only the relevant departments according to our consultations with experts in order to minimize this limitation. Thirdly, we did not take into consideration people who repeatedly quit and relapse into smoking. Such an endeavor would require even more data (which is unavailable) and complex models; however, their exclusion may not significantly affect our results since this group is probably captured either in the former or current smoker group.

Fourthly, even though one may anticipate an increase in government revenue with an increase in tobacco taxes, this is not the case when the effect of reduced consumption (fewer units) outcrowds the effect of increased unit taxes. In this work, we did not undertake supplementary analyses to explore the net impact on government revenue. Fifth, given our choice of a governmental perspective, including changes in tax revenue in the estimation of the cost-effectiveness ratio could have been appropriate. Such an analysis might have impacted the ACER results either negatively or positively. The overall effect of the changes in tobacco revenue to the estimates of ACER remains an empirical question that depend on the price elasticity of tobacco consumption. Sixth, the inclusion of only two smoking-related diseases (IHD and stroke), even though they cover about 30% of the disease burden attributable to tobacco smoking, likely underestimates the health gains related to tobacco control, pulling in our results in the conservative direction. Lastly, we modelled our interventions as being independent of each other, i.e. one by one in the absence of other interventions. This implies that the selection of one intervention will not influence the costs or effectiveness of other, independent interventions. However, this assumption is questionable in this case due to competing risk reductions. If, for instance, tax reform is effectively introduced, the effect of the other interventions would be lower because the baseline risk is changed. Consequently the resulting individual cost-effectiveness ratios might therefore be optimistic when several are implemented simulateneously, although this might be countered by economies of scale if several inititatives were coordinated and introduced as bundles of interventions.

## Conclusions

This study provides evidence on the cost and cost-effectiveness of tobacco control interventions in Tanzania. The model results showed that five population-based tobacco control strategies, namely: a ban on tobacco advertisements, package labelling of tobacco products, smoke-free environments, mass media campaigns and tobacco taxation, offer good value for money in the primary prevention of CVD in Tanzania. Despite these interventions being very cost-effective, they should not automatically be recommended for implementation, but the evidence should form a basis for discussion in the policy agenda to promote population health in Tanzania. Additionally, a budget impact analysis should be conducted to assess the government’s ability to implement these interventions.

## Supporting information

S1 Text Calculations of number of never, current and former smokers. (DOCX)

S2 Text Detailed description of tobacco control interventions modelled. (DOCX)

S3 Text Intervention effects on smoking prevalence. (DOCX)

S4 Text Calculation of health outcome. (DOCX)

S1 Table Cost breakdown for tobacco control measures. (DOCX)
