# Supplementary material for: Cost-effectiveness analysis of population-based tobacco control strategies in the prevention of cardiovascular diseases in Tanzania
Source: PLoS One. 2017 Aug 2;12(8):e0182113. doi: 10.1371/journal.pone.0182113 (PMC5540531; doi:10.1371/journal.pone.0182113)
Supplement: S1 Text — (DOCX) [file pone.0182113.s001.docx]

**S1 Text: Calculations of number of never, current and former smokers**

${1.Never}_{t,x}={Never}_{t-1,x-1} X \left( 1-p_{t-1,x-1}^{never} \right)X (1-I_{x})$

In case of no cessation of smoking ($C_{x}=0)$;

${2a. Current}_{t,x}={Current}_{t-1,x-1} X \left( 1-p_{t-1,x-1}^{current} \right)+{Never}_{t-1,x-1} X \left( 1-p_{t-1,x-1}^{never} \right)X I_{x}$

In case of no initiation to smoking ($I_{x}=0)$;

${2b. Current}_{t,x}={Current}_{t-1,x-1} X \left( 1-p_{t-1,x-1}^{current} \right)X (1-C_{x})$

$${3. Former}_{t,x}={Former}_{t-1,x-1} X \left( 1-p_{t-1,x-1}^{former} \right)+{Former}_{t-1,x-1} X \left( 1-p_{t-1,x-1}^{former} \right)X C_{x}$$

For the age 0 and 15, the formulae used were as follows:

$${Never}_{t,0,s}=\sum_{k=0}^{n} \left( {Female}_{t-1.a} \right) X {Fertility}_{t,x} X {Sex ratio}_{t,s}$$

${Never}_{t,15}={Never}_{t-1,14} X \left( 1-p_{t-1,14}^{never} \right)X (1-{Prev}_{t,15}^{current})$

${Current}_{t,15}={Never}_{t-1,14} X \left( 1-p_{t-1,14}^{never} \right)X {Prev}_{t,15}^{current}$

Where:

${Never}_{t,x,s}$ is the number of never smokers at age *x*, year *t* and sex *s*

${Current}_{t,x},{Former}_{t,x}$ is the number of current and former smokers at age *x*, year *t*

*I_x_* is the proportion of never smokers who initiate smoking at age *x*

*C_x_* is the proportion of current smokers who cease smoking at age *x*

${Female}_{t,x}$ is the number of females at age *x*, year *t*

${Fertility}_{t,x}$ is the age-specific fertility rate at age *x*, year *t*

${Sex ratio}_{t,s}$ is the sex ratio at birth in year *t*
