# Supplementary material for: Cost-effectiveness analysis of population-based tobacco control strategies in the prevention of cardiovascular diseases in Tanzania
Source: PLoS One. 2017 Aug 2;12(8):e0182113. doi: 10.1371/journal.pone.0182113 (PMC5540531; doi:10.1371/journal.pone.0182113)
Supplement: S2 Text — (DOCX) [file pone.0182113.s002.docx]

**S2 Text: Detailed description of tobacco control interventions modelled.**

***Advertisement, promotion and sponsorship bans***

Tanzania has no comprehensive ban on the advertisement, promotion or sponsorship of tobacco products. The Tobacco Regulation Act (TPRA) specifically bans these activities on radio and television, but interpretations of how the law applies to domestic print media are conflicting [1, 2]. Even with the recent tobacco regulations, which make explicit twelve channels from which advertisements are prohibited, event sponsorship and promotional activities that do not target minors are allowable [3]. We analyze the effects of a comprehensive advertisement, promotion and sponsorship ban by including the costs of initial investment in revising the legislation, promotion and advocacy in the first year and further sensitization and training in the third year, as well as five years of ongoing management and law-enforcement activities.

***Package labelling of tobacco products***

The current tobacco act requires one of ten authorized warnings/messages to be displayed at any given time with no graphic display. The act does not specify the size, format or placement of the health warning on the tobacco product. One study showed that the warnings currently cover only 6% of the face of the pack, which is far from the recommended 30–50% principle display areas according to article 11 of the WHO’s FCTC [2, 4, 5]. For this analysis, this strategy was defined as both text and graphic messages covering 30% of the pack surface. The scope of the intervention included initial investment in revising the legislation, promotion and advocacy in the first year and further sensitization and training in the third year, as well as five years of ongoing management and law-enforcement activities.

***Smoke-free public places***

Smoking at work and in public places is prohibited in Tanzania. A “public place” is defined to include health-care establishments, libraries, places of worship, enclosed premises intended for socio-cultural meetings, sporting or recreational activities, public eating places, office buildings, public transport by air, land or sea, pavilions, enclosed environments such as markets and malls, and any other enclosed place to which the public has admittance [3, 4]. However, this measure as currently presented in Tanzania’s tobacco act and regulation is not FCTC-compliant in that it allows for designated smoking areas in public places and its enforcement is mostly non-existent [1, 4, 5]. This intervention’s scope entailed initial investment in revising the legislation, promotion and advocacy in the first year and further sensitization and training in the third year, as well as five years of ongoing management and law-enforcement activities. Different scenarios pertaining to the cost of non-smoking signs were modelled; in our base-case scenario, entities were assumed to be required by the act to pay for the non-smoking signs [4].

***Mass media campaigns***

Article 12 of the WHO’s FCTC stipulates education, communication and public awareness as a strategy to reduce the demand for tobacco products; however, Tanzania’s current TPRA is silent on this matter [4, 5]. We defined this intervention as the implementation of a number of mass media campaigns over five years. Such media included television, radio, newspapers, directories, the internet and exhibitions. The scope of the intervention included the development of promotional and educational materials, a tobacco control program website and overall management and operational activities over five years.

***Increasing the taxation on tobacco products***

Tanzania has no meaningful tobacco-specific taxation [1], and according to the Tanzania Revenue Authority, the excise tax rate on the price of cigarettes was 35% in 2013. This rate is one of the lowest in the SSA region and much lower than the 70% rate proposed by the WHO [5]. In this intervention we analyzed two scenarios that involved a tax increase: first from 35% to 50% (base case), and then from 35% to the maximum proposed by the WHO of 70%. Activities included are initial investments in revision of the legislation, media advocacy during the first year, further sensitization and training in the third year and management and monitoring activities over five years.

**References**

1. Drope J. Tobacco Control in Africa: People, Politics, and Policies: Anthem Press; 2011.

2. Sussman S, Pokhrel P, Black D, Kohrman M, Hamann S, Vateesatokit P, et al. Tobacco control in developing countries: Tanzania, Nepal, China, and Thailand as examples. Nicotine & Tobacco Research. 2007;9(Suppl 3):S447-S57.

3. Tobacco Products Regulations, (2014).

4. United Republic of Tanzania. THE TOBACCO PRODUCTS (REGULATION) ACT. Dar Es Salaam: Parliament of the United Republic of Tanzania, 2003.

5. World Health Organization. FRAMEWORK CONVENTION ON TOBACCO CONTROL. Geneva: 2003.
