# Supplementary material for: Cost-effectiveness analysis of population-based tobacco control strategies in the prevention of cardiovascular diseases in Tanzania
Source: PLoS One. 2017 Aug 2;12(8):e0182113. doi: 10.1371/journal.pone.0182113 (PMC5540531; doi:10.1371/journal.pone.0182113)
Supplement: S3 Text — (DOCX) [file pone.0182113.s003.docx]

**S4 Text: Intervention effects on smoking prevalence**

In order to affect smoking initiation or cessation behaviours, intervention effects were modelled as follows:

$$I_{x}^{after}={RR}_{initiation}^{effect} x I_{x}^{before}$$

$$C_{x}^{after}={RR}_{cessation}^{effect} x C_{x}^{before}$$

where

$I_{a}^{before, after}$ is the proportion of never smokers who initiated smoking at age x.

$C_{a}^{before, after}$ is the proportion of current smokers who cease smoking at age x.

${RR}_{initiation, cessation}^{effect}$ is the relative risk of intervention effect on smoking initiation or cessation rate.

Intervention effect for tobacco tax increase did not follow the above approach; rather it was as follows due to its one off impact on the base year.

$${Prop}_{2012, x}^{current}=[{Prop}_{2012, x-1}^{current} X {(1- p}_{2012, x-1}^{current})+{Prop}_{2012, x-1}^{never} X {(1- p}_{2012, x-1}^{never}) XI_{x}^{before, after}] X (1+ {Price}_{increase} X {Elasticity}_{participate})$$

in case C_x_ = 0

$${Prop}_{2012, x}^{current}=\left[ {Prop}_{2012, x-1}^{current} X {(1- p}_{2012, x-1}^{current} \right) X {(1- C}_{x}^{before})] X (1+{Price}_{increase} X {Elasticity}_{participate})$$

in case I_x_ = 0

where

${Prop}_{2012, x}^{current}$ is the proportion of current smoker at age x, in the year 2012.

$p_{2012, x-1}^{current}$ is the probability of dying of current smoker one year before age x, in the year 2012.

$Price\_increase$ is the propotional increase of consumer price of cigarettes at the population.

${Elasticity}_{participate}$is the price elasticity for smoking participation.

For continuation effect for the 10 years time horizon smoking initiation was modelled as:

$$I_{x}^{after}= I_{x}^{before} X (1+{Price}_{increase} X {Elasticity}_{initiation})$$

where

${Elasticity}_{initiation}$ is the price eleaticity for smoking initiation.
