# Supplementary material for: Cost-effectiveness analysis of population-based tobacco control strategies in the prevention of cardiovascular diseases in Tanzania
Source: PLoS One. 2017 Aug 2;12(8):e0182113. doi: 10.1371/journal.pone.0182113 (PMC5540531; doi:10.1371/journal.pone.0182113)
Supplement: S4 Text — (DOCX) [file pone.0182113.s004.docx]

**S5 Text: Calculation of health outcomes**

DALYs without and with intervention were calculated from prevalent years lived with disability (pYLD), which is the number of healthy years lost due to disability in the population based on prevalent cases, disability weights (see Table A in S5 Text) and the number of individuals in a cohort, as shown in the formula below:

DALYS_noint_ = (No of people in the Cohort_noint_) x (1-PrevCVD_noint_) x [1 - {1 - (PYLD-CVD_dw_)}] + (No of people in the Cohort_noint_) x PrevIHD_noint_ x [1 - {1 - (PYLD-CVD_dw_)}] x (IHD_dw_)] + (No of people in the Cohort_noint_) x PrevStroke_noint_ x [1 - {1 - (PYLD-CVD_dw_)}] x (Stroke_dw_)]

DALYS_int_ = (No of people in the Cohort_int_) x (1-PrevCVD_int_) x [1 - {1 - (PYLD-CVD_dw_)}] + (No of people in the Cohort_int_) x PrevIHD_int_ x [1 - {1 - (PYLD-CVD_dw_)}] x (IHD_dw_)] + (No of people in the Cohort_int_) x PrevStroke_int_ x [1 - {1 - (PYLD-CVD_dw_)}] x (Stroke_dw_)]

DALYS averted = DALYS_noint_ - DALYS_int_

where:

noint=no intervention, Prev=prevalence, PYLD=prevalent years lived with disability, IHD=ischemic heart disease, dw=disability weight, int=intervention

| **Table A: Age-specific and sex-specific disability weights and prevalent years lived with disability** | | | | | | |
| --- | --- | --- | --- | --- | --- | --- |
|  | Ischemic heart disease | | Stroke | | PYLD | |
| Age | Males | Females | Males | Females | Males | Females |
| 15 | 0.11664 | 0.11311 | 0.16865 | 0.16855 | 0.06846 | 0.07882 |
| 20 | 0.11781 | 0.11532 | 0.16872 | 0.16874 | 0.08243 | 0.09730 |
| 25 | 0.11845 | 0.11687 | 0.16846 | 0.16858 | 0.09563 | 0.11075 |
| 30 | 0.11658 | 0.11479 | 0.16820 | 0.16840 | 0.10971 | 0.12754 |
| 35 | 0.00799 | 0.00685 | 0.16717 | 0.16838 | 0.12779 | 0.14436 |
| 40 | 0.03017 | 0.02491 | 0.16195 | 0.16515 | 0.14316 | 0.15642 |
| 45 | 0.04134 | 0.03457 | 0.15750 | 0.15957 | 0.15490 | 0.16288 |
| 50 | 0.04941 | 0.04068 | 0.15570 | 0.15657 | 0.16301 | 0.16568 |
| 55 | 0.05366 | 0.04569 | 0.15535 | 0.15565 | 0.17301 | 0.17572 |
| 60 | 0.05501 | 0.04740 | 0.15318 | 0.15465 | 0.18697 | 0.18986 |
| 65 | 0.05518 | 0.04673 | 0.15259 | 0.15356 | 0.20396 | 0.20557 |
| 70 | 0.05489 | 0.04647 | 0.14871 | 0.15074 | 0.22559 | 0.22580 |
| 75 | 0.05133 | 0.04391 | 0.14828 | 0.14849 | 0.24666 | 0.24692 |
| 80+ | 0.04825 | 0.04582 | 0.14329 | 0.14074 | 0.31180 | 0.32669 |

PYLD=Prevalent years lived with disability
