# Supplementary material for: Cost-effectiveness analysis of population-based tobacco control strategies in the prevention of cardiovascular diseases in Tanzania
Source: PLoS One. 2017 Aug 2;12(8):e0182113. doi: 10.1371/journal.pone.0182113 (PMC5540531; doi:10.1371/journal.pone.0182113)
Supplement: S1 Table — (DOCX) [file pone.0182113.s005.docx]

**S3 Table: Cost breakdown for tobacco control measures**

**Table A: Intervention cost of advertisement, promotion and sponsorship bans**

|  | **Price (US$)** | **Quantity** | **Year 1** | **Year 2** | **Year 3** | **Year 4** | **Year 5** |
| --- | --- | --- | --- | --- | --- | --- | --- |
| 1. **Strategy development and evaluation** |  |  |  |  |  |  |  |
| **A.1. Law development** *(based on cost of developing TFDA law: Tshs 220 million). Tobacco control law will have 3 parts: advertisement ban, package labelling & smoking ban. Allocation: cost is equally divided between these 3 components* | 148,037 | 1*0.33 | 49,346 | 0% | 0% | 0% | 0% |
| **A.2. Decree development** *(to guide the implementation of the law) (same basis as above)* | 10,093 | 1*0.33 | 3,364 | 0% | 0% | 0% | 0% |
| **Sub-total A** |  |  | **52,710** | **0** | **0** | **0** | **0** |
| 1. **Human resources** |  |  |  |  |  |  |  |
| **B.1. National-level program management** |  |  |  |  |  |  |  |
| **Steering committee** *(9 members, no salary, meeting allowance only (Tshs 200,000 per person), 2 meetings/year). Allocation: 20% for the preventive section (of which 7.5% is for advertisement ban)* | 135 | 9*2*0.075 | 182 | 100% | 100% | 100% | 100% |
| **Director** (*Gross salary and other allowances) Allocation: 30% for the preventive section(of which 10% for advertisement ban)* | 3,257 | 1*12*0.1 | 3,908 | 100% | 100% | 100% | 100% |
| **Preventive section manager** (*Gross salary and other allowances) Allocation: 75% for the preventive section (of which 25% for advertisement ban)* | 2,523 | 1*12*0.25 | 7,569 | 100% | 100% | 100% | 100% |
| **Program officers** (*Gross salary and other allowances)* *Allocation: 100% for the preventive section of which 35% for advertisement ban* | 1,174 | 9*12*0.35 | 44,377 | 100% | 100% | 100% | 100% |
| **Director’s secretary** (*Gross salary and other allowances*). *Allocation as Director* | 483 | 1*12*0.1 | 580 | 100% | 100% | 100% | 100% |
| **Preventive section manager’s secretary** (*Gross salary and other allowances*). *Allocation as preventive section manager* | 483 | 1*12*0.25 | 1,449 | 100% | 100% | 100% | 100% |
| **Public relations** | Various | Various | 4,108 | 0% | 100% | 0% | 0% |
| **B.2. District-level program management** |  |  |  |  |  |  |  |
| **Steering committee** **– district level** (*7 CHMT, 169 districts, no allowance only refreshments, Tshs 5000/person, 1 meeting/month). Allocation: Discusses all district issues and tobacco control takes up 6% (divided equally among the 3 preventive components)* | 3 | 169*7*12*0.02 | 852 | 100% | 100% | 100% | 100% |
| **Steering committee** **– community level** *(Community meeting, quarterly, Ward Executive representatives (2554 wards) & 2 district representatives, Tshs 40,000/person and refreshments. Allocation: Discusses all community issues and tobacco control takes up 3% (divided equally among the 3 preventive components)* | 27 | 2892*4*0.01 | 3,123 | 100% | 100% | 100% | 100% |
| **B.3. Law enforcement and inspection** |  |  |  |  |  |  |  |
| **Lawyer** (Gross salary and other allowances) A*llocation 15% for advertisement ban)* | 985 | 1*12*0.15 | 1,773 | 100% | 100% | 100% | 100% |
| **Legal officer** (*Gross salary and other allowances*) Allocation as lawyer | 647 | 1*12*0.15 | 1,165 | 100% | 100% | 100% | 100% |
| **Field trips** Allowances: (*use on average 4 “mgambos”; per diem Tshs 5000/day, 2 inspectors; per diem Tshs 60,000/day, 1 driver; per diem Tshs 40,000, 20lts fuel/day @ Tshs 2,000/l), 4 routine inspections of 3 days duration for a sample of 85 districts and 2 days in transit)* | Various | Various | 242,512 | 100% | 100% | 100% | 100% |
| **Sub-total B** |  |  | **311,598** | **307,490** | **311,598** | **307,490** | **307,490** |
| 1. **Promotion, media and advocacy** |  |  |  |  |  |  |  |
| **Meetings -** *law dissemination to government officers and institutions, NGOs in Dar and 5 zones, 50 participants and 10 facilitators. Allocation: 90% distributed equally among the 3 preventive strategies* | 69,880 | 1*0.3 | 20,964 | 0% | 0% | 0% | 0% |
| **Press conference: In house,** 40 participants from *different national newspapers and 3 international newspapers/agencies (participant allowance: Tshs 20,000/person, coffee 5,000 per person, transport 10,000/person; facilitators 10). Allocation as meetings* | 976 | 3*0.3 | 878 | 0% | 0% | 0% | 0% |
| **Press conference: Mbeya, Mwanza & Arusha,** 54 participants from *different national newspapers (participants allowance varied: Tshs 80,000; 65,000 and 40,000/person depending on salary scale, coffee 5,000; transport 10,000/person; facilitators 5, air tickets & venue). Allocation as meetings* | 8,374 | 3*0.3 | 7,537 | 0% | 0% | 0% | 0% |
| **Key law messages -** *government institutions & gazette. Allocation as meetings* | 10,093 | 1*0.3 | 3,028 | 0% | 0% | 0% | 0% |
| **Orientation, sensitization and training.** *Carried out in the 1st and 3rd year*, *Allocation as meetings* | 3,743 | 30*0.33 | 37,430 | 0% | 100% | 0% | 0% |
| **Sub-total C** |  |  | **69,837** | **0** | **37,430** | **0** | **0** |
| 1. **Program supplies** |  |  | 0 | 0 | 0 | 0 | 0 |
| **Sub-total D** |  |  | **0** | **0** | **0** | **0** | **0** |
| 1. **Office rent, equipment and supplies,** *Allocation as human resources* | Various | Various | 6,547 | 5,679 | 6,547 | 5,679 | 5,679 |
| **Sub-total E** |  |  | **6,547** | **5,679** | **6,547** | **5,679** | **5,679** |
| 1. **Administration** *(Allocation 20% for advertisement ban)* |  |  |  |  |  |  |  |
| **F.1. Human resources – National level** |  |  |  |  |  |  |  |
| **Steering committee;** *Allocation 50% operations* | 135 | 9*2*0.5 | 1,215 | 100% | 100% | 100% | 100% |
| **Director;** *Allocation 40% operations* | 3,257 | 1*12*0.4 | 15,634 | 100% | 100% | 100% | 100% |
| **Operation section manager** *(Gross salary and other allowances) Allocation: 100%* | 2,523 | 1*12 | 30,276 | 100% | 100% | 100% | 100% |
| **Preventive and Education section managers** (Gross salary and other allowances) *Allocation 25% operations* | 2,523 | 2*12*0.25 | 15,138 | 100% | 100% | 100% | 100% |
| **Senior administrative officer** (*Gross salary and other allowances)* *Allocation 100% operations* | 1,537 | 1*12 | 18,444 | 100% | 100% | 100% | 100% |
| **Administrative officers** (*Gross salary and other allowances)* Allocation 100% | 647 | 2*12 | 15,528 | 100% | 100% | 100% | 100% |
| **Chief accountant** (*Gross salary and other allowances)*  Allocation 100% | 2,268 | 1*12 | 27,216 | 100% | 100% | 100% | 100% |
| **Accountants** (*Gross salary and other allowances)* Allocation 100% | 647 | 2*12 | 15,528 | 100% | 100% | 100% | 100% |
| **Senior procurement officer** (*Gross salary and other allowances*) Allocation 100% | 1,174 | 1*12 | 14,088 | 100% | 100% | 100% | 100% |
| **Procurement officer** (*Gross salary and other allowances)* Allocation 100% | 647 | 1*12 | 7,764 | 100% | 100% | 100% | 100% |
| **Planning officer** (*Gross salary and other allowances*) A*llocation 100%* | 911 | 1*12 | 10,932 | 100% | 100% | 100% | 100% |
| **Lawyer**; *Allocation 30%* | 985 | 1*12*0.3 | 3,546 | 100% | 100% | 100% | 100% |
| **Legal officer**; *Allocation as lawyer* | 647 | 1*12*0.3 | 2,329 | 100% | 100% | 100% | 100% |
| **Senior IT computing officer** (*Gross salary and other allowances)* A*llocation 100%* | 911 | 1*12 | 10,932 | 100% | 100% | 100% | 100% |
| **IT computing officer** (*Gross salary and other allowances*) Allocation 100% | 647 | 2*12 | 15,528 | 100% | 100% | 100% | 100% |
| **Operations manager’s secretary** (*Gross salary and other allowances*). *Allocation as Operations manager* | 483 | 1*12 | 5,796 | 100% | 100% | 100% | 100% |
| **Director’s secretary;** *Allocation as director* | 483 | 1*12*0.4 | 2,318 | 100% | 100% | 100% | 100% |
| **Prevention and Education managers’ secretary;** *Allocation as corresponding managers* | 483 | 2*12*0.25 | 2,898 | 100% | 100% | 100% | 100% |
| **Receptionist** (*Gross salary and other allowances*) Allocation 100% | 483 | 1*12 | 5,796 | 100% | 100% | 100% | 100% |
| **Drivers** (*Gross salary and other allowances*) Allocation 100% | 306 | 2*12 | 7,344 | 100% | 100% | 100% | 100% |
| **Cleaners** (*Gross salary and other allowances*) Allocation 100% | 306 | 4*12 | 14,688 | 100% | 100% | 100% | 100% |
| **Human resources - Zonal offices** | Various | Various | 261,897 | 100% | 100% | 100% | 100% |
| **Law enforcement and inspection – Zonal level** | Various | Various | 90,370 | 100% | 100% | 100% | 100% |
| **F.2. Promotion and advocacy – Zonal offices** | Various | Various | 225,761 | 112,880 | 225,761 | 112,880 | 112,880 |
| **F.3. Program supplies** |  |  | 0 | 100% | 100% | 100% | 100% |
| **F.4. Rent, equipment and office supplies** | Various | Various | 79,560 | 100% | 100% | 100% | 100% |
| **F.5. Utilities** | Various | Various | 89,310 | 41,615 | 41,615 | 47,070 | 41,615 |
| **Sub-total F** |  | 1*0.20 | **197,967** | **165,852** | **188,428** | **166,943** | **165,852** |
| **TOTAL** |  |  | **638,659** | **479,021** | **544,003** | **480,112** | **479,021** |

**Table B: Intervention cost of package labelling of tobacco products**

|  | **Price (US$)** | **Quantity** | **Year 1** | **Year 2** | **Year 3** | **Year 4** | **Year 5** |
| --- | --- | --- | --- | --- | --- | --- | --- |
| 1. **Strategy development and evaluation** |  |  |  |  |  |  |  |
| **A.1. Law development** *(based on cost of developing TFDA law: Tshs 220 million). Tobacco control law will have 3 parts: advertisement ban, package labelling & smoking ban. Allocation: cost is equally divided between these 3 components* | 148,037 | 1*0.33 | 49,346 | 0% | 0% | 0% | 0% |
| **A.2. Decree development** *(to guide the implementation of the law) (same basis as above)* | 10,093 | 1*0.33 | 3,364 | 0% | 0% | 0% | 0% |
| **Sub-total A** |  |  | **52,710** | **0** | **0** | **0** | **0** |
| 1. **Human resources** |  |  |  |  |  |  |  |
| **B.1. National-level program management** |  |  |  |  |  |  |  |
| **Steering committee** *(9 members, no salary, meetings allowance only (Tshs 200,000 per person), 2 meetings/year). Allocation: 20% for the preventive section (of which 5% is for package labelling).* | 135 | 9*2*0.05 | 122 | 100% | 100% | 100% | 100% |
| **Director** (*Gross salary and other allowances) Allocation: 30% for the preventive section(of which 10% for package labelling* | 3,257 | 1*12*0.1 | 3,908 | 100% | 100% | 100% | 100% |
| **Preventive section manager** (*Gross salary and other allowances) Allocation: 75% for the preventive section (of which 25% for package labelling* | 2,523 | 1*12*0.25 | 7,569 | 100% | 100% | 100% | 100% |
| **Program officers** (*Gross salary and other allowances)* *Allocation: 100% for the preventive section of which 30% for package labelling* | 1,174 | 9*12*0.3 | 38,038 | 100% | 100% | 100% | 100% |
| **Director’s secretary** (*Gross salary and other allowances*). *Allocation as Director* | 483 | 1*12*0.1 | 580 | 100% | 100% | 100% | 100% |
| **Preventive section manager’s secretary** (*Gross salary and other allowances*). *Allocation as preventive section manager* | 483 | 1*12*0.25 | 1,449 | 100% | 100% | 100% | 100% |
| **Public relations** | Various | Various | 4,108 | 0% | 100% | 0% | 0% |
| **B.2. District-level program management** |  |  |  |  |  |  |  |
| **Steering committee** **– district level** (*7 CHMT, 169 districts, no allowance only refreshments Tshs 5000/person, 1 meeting/month). Allocation: Discusses all district issues and tobacco control takes up 6% (divided equally among the 3 preventive components)* | 3 | 169*7*12*0.02 | 852 | 100% | 100% | 100% | 100% |
| **Steering committee** **– community level** *(Community meeting, quarterly, Ward Executive representatives (2554 wards) & 2 district representatives, Tshs 40,000/person and refreshments Allocation: Discusses all community issues and tobacco control takes up 3% (divided equally among the 3 preventive components)* | 27 | 2892*4*0.01 | 3,123 | 100% | 100% | 100% | 100% |
| **B.3. Law enforcement and inspection** |  |  |  |  |  |  |  |
| **Lawyer** (Gross salary and other allowances) A*llocation 15% for package labelling)* | 985 | 1*12*0.15 | 1,773 | 100% | 100% | 100% | 100% |
| **Legal officer** (*Gross salary and other allowances*) Allocation as lawyer | 647 | 1*12*0.15 | 1,165 | 100% | 100% | 100% | 100% |
| **Field trips** Allowances: (*use on average 4 “mgambos”; per diem Tshs 5000/day, 2 inspectors; per diem Tshs 60,000/day, 1 driver; per diem Tshs 40,000, 20lts fuel/day @ Tshs 2,000/l), 4 routine inspections of 3 days duration for a sample of 85 districts and 2 days in transit.* | Various | Various | 242,512 | 100% | 100% | 100% | 100% |
| **Sub-total B** |  |  | **305,199** | **301,151** | **305,199** | **301,091** | **301,091** |
| 1. **Promotion, media and advocacy** |  |  |  |  |  |  |  |
| **Meetings -** *law dissemination to government officers and institutions, NGOs in Dar and 5 zones, 50 participants and 10 facilitators. Allocation: 90% distributed equally among the 3 preventive strategies* | 69,880 | 1*0.3 | 20,964 | 0% | 0% | 0% | 0% |
| **Press conference: In house,** 40 participants from *different national newspapers and 3 international newspapers/agencies (participants allowance: Tshs 20,000/person, coffee 5,000 per person, transport 10,000/person; facilitators 10). Allocation as meetings* | 976 | 3*0.3 | 878 | 0% | 0% | 0% | 0% |
| **Press conference: Mbeya, Mwanza & Arusha,** 54 participants from *different national newspapers (participants allowance varied: Tshs 80,000; 65,000 and 40,000/person depending on salary scale, coffee 5,000, transport 10,000/person; facilitators 5, air tickets & venue). Allocation as meetings* | 8,374 | 3*0.3 | 7,537 | 0% | 0% | 0% | 0% |
| **Key law messages -** *government institutions & gazette. Allocation as meetings* | 10,093 | 1*0.3 | 3,028 | 0% | 0% | 0% | 0% |
| **Orientation, sensitization and training.** *Done in the 1st and 3rd year*, *Allocation as meetings* | 3,743 | 30*0.33 | 37,430 | 0% | 100% | 0% | 0% |
| **Sub-total C** |  |  | **69,837** | **0** | **37,430** | **0** | **0** |
| 1. **Program supplies** |  |  | 0 | 0 | 0 | 0 | 0 |
| **Sub-total D** |  |  | **0** | **0** | **0** | **0** | **0** |
| 1. **Office rent, equipment and supplies** *Allocation as human resource* | Various | Various | 6,278 | 5,409 | 6,278 | 5,409 | 5,409 |
| **Sub-total E** |  |  | **6,278** | **5,409** | **6,278** | **5,409** | **5,409** |
| 1. **Administration** *(Allocation 20% for package labelling)* |  |  |  |  |  |  |  |
| **F.1. Human resources – National level** |  |  |  |  |  |  |  |
| **Steering committee;** *Allocation 50% operations* | 135 | 9*2*0.5 | 1,215 | 100% | 100% | 100% | 100% |
| **Director;** *Allocation 40% operations* | 3,257 | 1*12*0.4 | 15,634 | 100% | 100% | 100% | 100% |
| **Operation section manager** *(Gross salary and other allowances) Allocation: 100%* | 2,523 | 1*12 | 30,276 | 100% | 100% | 100% | 100% |
| **Preventive and Education section managers** (Gross salary and other allowances) *Allocation 25% operations* | 2,523 | 2*12*0.25 | 15,138 | 100% | 100% | 100% | 100% |
| **Senior administrative officer** (*Gross salary and other allowances)* *Allocation 100% operations* | 1,537 | 1*12 | 18,444 | 100% | 100% | 100% | 100% |
| **Administrative officers** (*Gross salary and other allowances)* Allocation 100% | 647 | 2*12 | 15,528 | 100% | 100% | 100% | 100% |
| **Chief accountant** (*Gross salary and other allowances)*  Allocation 100% | 2,268 | 1*12 | 27,216 | 100% | 100% | 100% | 100% |
| **Accountants** (*Gross salary and other allowances)* Allocation 100% | 647 | 2*12 | 15,528 | 100% | 100% | 100% | 100% |
| **Senior procurement officer** (*Gross salary and other allowances*) Allocation 100% | 1,174 | 1*12 | 14,088 | 100% | 100% | 100% | 100% |
| **Procurement officer** (*Gross salary and other allowances)* Allocation 100% | 647 | 1*12 | 7,764 | 100% | 100% | 100% | 100% |
| **Planning officer** (*Gross salary and other allowances*) A*llocation 100%* | 911 | 1*12 | 10,932 | 100% | 100% | 100% | 100% |
| **Lawyer**; *Allocation 30%* | 985 | 1*12*0.3 | 3,546 | 100% | 100% | 100% | 100% |
| **Legal officer**; *Allocation as lawyer* | 647 | 1*12*0.3 | 2,329 | 100% | 100% | 100% | 100% |
| **Senior IT computing officer** (*Gross salary and other allowances)* A*llocation 100%* | 911 | 1*12 | 10,932 | 100% | 100% | 100% | 100% |
| **IT computing officer** (*Gross salary and other allowances*) Allocation 100% | 647 | 2*12 | 15,528 | 100% | 100% | 100% | 100% |
| **Operations manager’s secretary** (*Gross salary and other allowances*). *Allocation as Operations manager* | 483 | 1*12 | 5,796 | 100% | 100% | 100% | 100% |
| **Director’s secretary;** *Allocation as director* | 483 | 1*12*0.4 | 2,318 | 100% | 100% | 100% | 100% |
| **Prevention and Education managers’ secretary;** *Allocation as corresponding managers* | 483 | 2*12*0.25 | 2,898 | 100% | 100% | 100% | 100% |
| **Receptionist** (*Gross salary and other allowances*) Allocation 100% | 483 | 1*12 | 5,796 | 100% | 100% | 100% | 100% |
| **Drivers** (*Gross salary and other allowances*) Allocation 100% | 306 | 2*12 | 7,344 | 100% | 100% | 100% | 100% |
| **Cleaners** (*Gross salary and other allowances*) Allocation 100% | 306 | 4*12 | 14,688 | 100% | 100% | 100% | 100% |
| **Human resources - Zonal offices** | Various | Various | 261,897 | 100% | 100% | 100% | 100% |
| **Law enforcement and inspection – Zonal level** | Various | Various | 90,370 | 100% | 100% | 100% | 100% |
| **F.2. Promotion and advocacy – Zonal offices** | Various | Various | 225,761 | 112,880 | 225,761 | 112,880 | 112,880 |
| **F.3. Program supplies** |  |  | 0 | 100% | 100% | 100% | 100% |
| **F.4. Rent, Equipment and office supplies** | Various | Various | 79,560 | 100% | 100% | 100% | 100% |
| **F.5. Utilities** | Various | Various | 89,310 | 41,615 | 41,615 | 47,070 | 41,615 |
| **Sub-total F** |  | 1*0.20 | **197,967** | **165,852** | **188,428** | **166,943** | **165,852** |
| **TOTAL** |  |  | **631,991** | **472,352** | **537,335** | **473,443** | **472,352** |

**Table C: Intervention cost of smoke ban in workplaces and public places**

|  | **Price (US$)** | **Quantity** | **Year 1** | **Year 2** | **Year 3** | **Year 4** | **Year 5** |
| --- | --- | --- | --- | --- | --- | --- | --- |
| 1. **Strategy development and evaluation** |  |  |  |  |  |  |  |
| **A.1. Law development** *(based on cost of developing TFDA law: Tshs 220 million). Tobacco control law will have 3 parts: advertisement ban, package labelling & smoking ban. Allocation: cost is equally divided in these 3 components* | 148,037 | 1*0.33 | 49,346 | 0% | 0% | 0% | 0% |
| **A.2. Decree development** *(to guide the implementation of the law) (same basis as above)* | 10,093 | 1*0.33 | 3,364 | 0% | 0% | 0% | 0% |
| **Sub-total A** |  |  | **52,710** | **0** | **0** | **0** | **0** |
| 1. **Human resources** |  |  |  |  |  |  |  |
| **B.1. National-level program management** |  |  |  |  |  |  |  |
| **Steering committee** *(9 members, no salary, meetings allowance only (Tshs 200,000 per person), 2 meetings/year). Allocation: 20% for the preventive section (of which 7.5% is for smoking ban)* | 135 | 9*2*0.075 | 182 | 100% | 100% | 100% | 100% |
| **Director** (*Gross salary and other allowances) Allocation: 30% for the preventive section (of which 10% for smoking ban)* | 3,257 | 1*12*0.1 | 3,908 | 100% | 100% | 100% | 100% |
| **Preventive section manager** (*Gross salary and other allowances) Allocation: 75% for the preventive section (of which 25% for smoking ban)* | 2,523 | 1*12*0.25 | 7,569 | 100% | 100% | 100% | 100% |
| **Program officers** (*Gross salary and other allowances)* *Allocation: 100% for the preventive section of which 35% for smoking ban* | 1,174 | 9*12*0.35 | 44,377 | 100% | 100% | 100% | 100% |
| **Director’s secretary** (*Gross salary and other allowances*). *Allocation as Director* | 483 | 1*12*0.1 | 580 | 100% | 100% | 100% | 100% |
| **Preventive section manager’s secretary** (*Gross salary and other allowances*). *Allocation as preventive section manager* | 483 | 1*12*0.25 | 1,449 | 100% | 100% | 100% | 100% |
| **Public relations** | Various | Various | 4,108 | 0% | 100% | 0% | 0% |
| **B.2. District-level program management** |  |  |  |  |  |  |  |
| **Steering committee** **– district level** (*7 CHMT, 169 districts, no allowance only refreshments Tshs 5000/person, 1 meeting/month) Allocation: Discusses all district issues and tobacco control takes up 6% (divided equally among the 3 preventive components)* | 3 | 169*7*12*0.02 | 852 | 100% | 100% | 100% | 100% |
| **Steering committee** **– community level** *(Community meeting, quarterly, Ward Executive representatives (2554 wards) & 2 district representatives, Tshs 40,000/person and refreshments Allocation: Discusses all community issues and tobacco control takes up 3% (divided equally among the 3 preventive components)* | 27 | 2892*4*0.01 | 3,123 | 100% | 100% | 100% | 100% |
| **B.3. Law enforcement and inspection** |  |  |  |  |  |  |  |
| **Lawyer** (Gross salary and other allowances) A*llocation 15% for smoking in public and work places ban)* | 985 | 1*12*0.15 | 1,773 | 100% | 100% | 100% | 100% |
| **Legal officer** (*Gross salary and other allowances*) Allocation as lawyer | 647 | 1*12*0.15 | 1,165 | 100% | 100% | 100% | 100% |
| **Day field trips** Allowances: (*use on average 4 “mgambos”; per diem Tshs 5000/day, 2 inspectors; per diem Tshs 60,000/day, 1 driver; per diem Tshs 40,000, 20lts fuel/day @ Tshs 2,000/l), 4 routine inspections of 3 days duration for a sample of 85 districts and 2 days in transit* | Various | Various | 242,512 | 100% | 100% | 100% | 100% |
| **Night field trips** Allowances: (*use on average 4 “mgambos”; per diem Tshs 1000/day, 2 inspectors; per diem Tshs 80,000/day, 1 driver; per diem Tshs 40,000, 20lts fuel/day @ Tshs 2,000/l), 4 routine inspections of 3 days duration for a sample of 25% of the 169 districts and 2 days in transit* | Various | Various | 159,880 | 100% | 100% | 100% | 100% |
| **Sub-total B** |  |  | **471,478** | **467,370** | **471,478** | **467,370** | **467,370** |
| 1. **Promotion, media and advocacy** |  |  |  |  |  |  |  |
| **Meetings -** *law dissemination to government officers and institutions, NGOs in Dar and 5 zones, 50 participants and 10 facilitators. Allocation: 90% distributed equally among the 3 preventive strategies* | 69,880 | 1*0.3 | 20,964 | 0% | 0% | 0% | 0% |
| **Press conference: In house,** 40 participants from *different national newspapers and 3 international newspapers/agencies (participants allowance: Tshs 20,000/person, coffee 5,000 per person, transport 10,000/person; facilitators 10). Allocation as meetings* | 976 | 3*0.3 | 878 | 0% | 0% | 0% | 0% |
| **Press conference: Mbeya, Mwanza & Arusha,** 54 participants from *different national newspapers (participants allowance varied: Tshs 80,000; 65,000 and 40,000/person depending on salary scale, coffee 5,000, transport 10,000/person; facilitators 5, air tickets & venue). Allocation as meetings* | 8,374 | 3*0.3 | 7,537 | 0% | 0% | 0% | 0% |
| **Key law messages -** *government institutions & gazette. Allocation as meetings* | 10,093 | 1*0.3 | 3,028 | 0% | 0% | 0% | 0% |
| **Orientation, sensitization and training.** *Carried out in the 1st and 3rd year*, *Allocation as meetings* | 3,743 | 30*0.33 | 37,430 | 0% | 100% | 0% | 0% |
| **Sub-total C** |  |  | **69,837** | **0** | **37,430** | **0** | **0** |
| 1. **Program supplies** |  |  |  |  |  |  |  |
| **Option 1 (Base case):** Cost of non-smoking sign is zero - *as law requires entities to pay for non-smoking signs posted in their venues.* |  |  | 0 | 0 | 0 | 0 | 0 |
| **Option 2:** Non-smoking signs - based *on population, 1 sign per 100 people; cost of hard-material signs: Tshs 20,000/sign, 10% replacement in the 4^th^ year.* | 13 | 45,000,000/100 | 5,850,000 | 0% | 0% | 0% | 585,000 |
| **Option 3:** Non-smoking signs - *based on average numbers of signs per ward/district/region and national agency (2554 wards with 80 signs per ward, 169 districts with 200 signs per district, 30 provinces with 300 signs per region, 150,000 government offices and enterprises - 4 signs each, 30 national units and ministries with 20 signs per unit) cost of hard-material signs: Tshs 20,000/sign, 10% replacement in the 4^th^ year* | 13 | 849,220 | 11,039,860 | 0% | 0% | 0% | 1,103,986 |
| **Option 4:** Non-smoking signs *- as paper sticker at cost Tshs 2000/sticker in locations mentions in option 2, 40% replacement in the 4^th^ year* | 1 | 849,220 | 849,220 | 0% | 0% | 0% | 339,688 |
| **Sub-total D - Option 1 (Base case)** |  |  | **0** | **0** | **0** | **0** | **0** |
| **Sub-total D - Option 2** |  |  | **5,850,000** | **0** | **0** | **585,000** | **0** |
| **Sub-total D - Option 3** |  |  | **11,039,860** | **0** | **0** | **1,103,986** | **0** |
| **Sub-total D - Option 4** |  |  | **849,220** | **0** | **0** | **339,688** | **0** |
| 1. **Office rent, equipment and supplies,** *Allocation as human resources* | Various | Various | 6,547 | 5,679 | 6,547 | 5,679 | 5,679 |
| **Sub-total E** |  |  | **6,547** | **5,679** | **6,547** | **5,679** | **5,679** |
| 1. **Administration** *(Allocation 20% for smoking ban)* |  |  |  |  |  |  |  |
| **F.1. Human resources – National level** |  |  |  |  |  |  |  |
| **Steering committee;** *Allocation 50% operations* | 135 | 9*2*0.5 | 1,215 | 100% | 100% | 100% | 100% |
| **Director;** *Allocation 40% operations* | 3,257 | 1*12*0.4 | 15,634 | 100% | 100% | 100% | 100% |
| **Operation section manager** *(Gross salary and other allowances) Allocation: 100%* | 2,523 | 1*12 | 30,276 | 100% | 100% | 100% | 100% |
| **Preventive and Education section managers** (Gross salary and other allowances) *Allocation 25% operations* | 2,523 | 2*12*0.25 | 15,138 | 100% | 100% | 100% | 100% |
| **Senior administrative officer** (*Gross salary and other allowances)* *Allocation 100% operations* | 1,537 | 1*12 | 18,444 | 100% | 100% | 100% | 100% |
| **Administrative officers** (*Gross salary and other allowances)* Allocation 100% | 647 | 2*12 | 15,528 | 100% | 100% | 100% | 100% |
| **Chief accountant** (*Gross salary and other allowances)*  Allocation 100% | 2,268 | 1*12 | 27,216 | 100% | 100% | 100% | 100% |
| **Accountants** (*Gross salary and other allowances)* Allocation 100% | 647 | 2*12 | 15,528 | 100% | 100% | 100% | 100% |
| **Senior procurement officer** (*Gross salary and other allowances*) Allocation 100% | 1,174 | 1*12 | 14,088 | 100% | 100% | 100% | 100% |
| **Procurement officer** (*Gross salary and other allowances)* Allocation 100% | 647 | 1*12 | 7,764 | 100% | 100% | 100% | 100% |
| **Planning officer** (*Gross salary and other allowances*) A*llocation 100%* | 911 | 1*12 | 10,932 | 100% | 100% | 100% | 100% |
| **Lawyer**; *Allocation 30%* | 985 | 1*12*0.3 | 3,546 | 100% | 100% | 100% | 100% |
| **Legal officer**; *Allocation as lawyer* | 647 | 1*12*0.3 | 2,329 | 100% | 100% | 100% | 100% |
| **Senior IT computing officer** (*Gross salary and other allowances)* A*llocation 100%* | 911 | 1*12 | 10,932 | 100% | 100% | 100% | 100% |
| **IT computing officer** (*Gross salary and other allowances*) Allocation 100% | 647 | 2*12 | 15,528 | 100% | 100% | 100% | 100% |
| **Operations manager’s secretary** (*Gross salary and other allowances*). *Allocation as Operations manager* | 483 | 1*12 | 5,796 | 100% | 100% | 100% | 100% |
| **Director’s secretary;** *Allocation as director* | 483 | 1*12*0.4 | 2,318 | 100% | 100% | 100% | 100% |
| **Prevention and Education managers’ secretary;** *Allocation as corresponding managers* | 483 | 2*12*0.25 | 2,898 | 100% | 100% | 100% | 100% |
| **Receptionist** (*Gross salary and other allowances*) Allocation 100% | 483 | 1*12 | 5,796 | 100% | 100% | 100% | 100% |
| **Drivers** (*Gross salary and other allowances*) Allocation 100% | 306 | 2*12 | 7,344 | 100% | 100% | 100% | 100% |
| **Cleaners** (*Gross salary and other allowances*) Allocation 100% | 306 | 4*12 | 14,688 | 100% | 100% | 100% | 100% |
| **Human resources - Zonal offices** | Various | Various | 261,897 | 100% | 100% | 100% | 100% |
| **Law enforcement and inspection – Zonal level** | Various | Various | 90,370 | 100% | 100% | 100% | 100% |
| **F.2. Promotion and advocacy – Zonal offices** | Various | Various | 225,761 | 112,880 | 225,761 | 112,880 | 112,880 |
| **F.3. Program supplies** |  |  | 0 | 100% | 100% | 100% | 100% |
| **F.4. Office rent, equipment and supplies,** *Allocation as human resource* | Various | Various | 79,560 | 100% | 100% | 100% | 100% |
| **F.5. Utilities** | Various | Various | 89,310 | 41,615 | 41,615 | 47,070 | 41,615 |
| **Sub-total F** |  | 1*0.20 | **197,967** | **165,852** | **188,428** | **166,943** | **165,852** |
| **TOTAL – Option 1 (Base case)** |  |  | **799,539** | **638,901** | **703,883** | **639,992** | **638,901** |
| **TOTAL – Option 2** |  |  | **6,649,539** | **638,901** | **703,883** | **1,224,992** | **638,901** |
| **TOTAL – Option 3** |  |  | **11,839,399** | **638,901** | **703,883** | **1,743,978** | **638,901** |
| **TOTAL – Option 4** |  |  | **1,648,759** | **638,901** | **703,883** | **979,680** | **638,901** |

**Table D: Intervention cost of mass media campaigns**

|  | **Price (US$)** | **Quantity** | **Year 1** | **Year 2** | **Year 3** | **Year 4** | **Year 5** |
| --- | --- | --- | --- | --- | --- | --- | --- |
| 1. **Strategy development and evaluation** |  |  | 0 | 0 | 0 | 0 | 0 |
| **Sub-total A** |  |  | **0** | **0** | **0** | **0** | **0** |
| 1. **Human resources** |  |  |  |  |  |  |  |
| **B.1. National-level program management** |  |  |  |  |  |  |  |
| **Steering committee** *(9 members, no salary, meetings allowance only (Tshs 200,000 per person), 2 meetings/year). Allocation: 10% for the education section* | 135 | 9*2*0.1 | 243 | 100% | 100% | 100% | 100% |
| **Director** (*Gross salary and other allowances) Allocation: 15% for the education section (of which 10% is for information and education)* | 3,257 | 1*12*0.1 | 3,908 | 100% | 100% | 100% | 100% |
| **Education section manager** (*Gross salary and other allowances) Allocation: 50% for information and education* | 2,523 | 1*12*0.5 | 15,138 | 100% | 100% | 100% | 100% |
| **Public relation officers** (*Gross salary and other allowances)* *Allocation: 80% for information and education* | 647 | 3*12*0.8 | 18,634 | 100% | 100% | 100% | 100% |
| **Director’s secretary** (*Gross salary and other allowances*). *Allocation as Director* | 483 | 1*12*0.1 | 580 | 100% | 100% | 100% | 100% |
| **Education manager’s secretary** (*Gross salary and other allowances*). *Allocation as education manager* | 483 | 1*12*0.5 | 2,898 | 100% | 100% | 100% | 100% |
| **Librarian** (*Gross salary and other allowances*). *Allocation 90%* | 772 | 1*12*0.9 | 8,338 | 100% | 100% | 100% | 100% |
| **Public relations** | Various | Various | 4,108 | 16,431 | 4,108 | 16,431 | 16,431 |
| **B.2. Law education** |  |  |  |  |  |  |  |
| **Lawyer** (Gross salary and other allowances) A*llocation 25% for law education)* | 985 | 1*12*0.25 | 2,955 | 100% | 100% | 100% | 100% |
| **Legal officer** (*Gross salary and other allowances*) Allocation as lawyer | 647 | 1*12*0.25 | 1,941 | 100% | 100% | 100% | 100% |
| **Sub-total B** |  |  | **58,748** | **71,066** | **58,748** | **71,066** | **71,066** |
| 1. **Promotion, media and advocacy** |  |  |  |  |  |  |  |
| **C.1. General promotion, media and advocacy** |  |  |  |  |  |  |  |
| **Meetings -** *law dissemination to government officers and institutions, NGOs in Dar and 5 zones, 50 participants and 10 facilitators. Allocation: 10% for education section* | 69,880 | 1*0.1 | 6,988 | 0% | 0% | 0% | 0% |
| **Press conference: In house,** 40 participants from *different national newspapers and 3 international newspapers/agencies (participants allowance: Tshs 20,000/person, coffee 5,000 per person, transport 10,000/person; facilitators 10). Allocation as meetings* | 976 | 3*0.1 | 293 | 0% | 0% | 0% | 0% |
| **Press conference: Mbeya, Mwanza & Arusha,** 54 participants from *different national newspapers (participants allowance varied: Tshs 80,000; 65,000 and 40,000/person depending on salary scale, coffee 5,000, transport 10,000/person; facilitators 5, air tickets & venue). Allocation as meetings* | 8,374 | 3*0.1 | 2,512 | 0% | 0% | 0% | 0% |
| **Key law messages -** *government institutions & gazette. Allocation as meetings* | 10,093 | 1*0.1 | 1,009 | 0% | 0% | 0% | 0% |
| **C.1. Specific promotion, media and advocacy** |  |  |  |  |  |  |  |
| **TV and Radio** | Various | Various | 61,130 | 100% | 100% | 100% | 100% |
| **Newspapers and directories** | Various | Various | 29,315 | 100% | 100% | 100% | 100% |
| **Promotional materials** | Various | Various | 14,641 | 100% | 100% | 100% | 100% |
| **Educational materials** | Various | Various | 15,950 | 100% | 100% | 100% | 100% |
| **Tobacco national control website** | Various | Various | 5,370 | 100% | 100% | 100% | 100% |
| **Exhibitions** | Various | Various | 19,268 | 100% | 100% | 100% | 100% |
| **Subtotal C** |  |  | **156,476** | **145,674** | **145,674** | **145,674** | **145,674** |
| 1. **Program supplies** |  |  | 0 | 0 | 0 | 0 | 0 |
| Annual report writing | Block figure | Block figure | 4,249 | 100% | 100% | 100% | 100% |
| Scientific books | Block figure | Block figure | 13,574 | 100% | 100% | 100% | 100% |
| **Sub-total D** |  |  | **17,823** | **17,823** | **17,823** | **17,823** | **17,823** |
| 1. **Office rent, equipment and supplies**. *Allocation as human resources* | Various | Various | 12,621 | 15,297 | 12,621 | 15,297 | 15,297 |
| **Sub-total E** |  |  | **12,621** | **15,297** | **12,621** | **15,297** | **15,297** |
| 1. **Administration** *(Allocation 20% for education section)* |  |  |  |  |  |  |  |
| **F.1. Human resources – National level** |  |  |  |  |  |  |  |
| **Steering committee;** *Allocation 50% operations* | 135 | 9*2*0.5 | 1,215 | 100% | 100% | 100% | 100% |
| **Director;** *Allocation 40% operations* | 3,257 | 1*12*0.4 | 15,634 | 100% | 100% | 100% | 100% |
| **Operation section manager** *(Gross salary and other allowances) Allocation: 100%* | 2,523 | 1*12 | 30,276 | 100% | 100% | 100% | 100% |
| **Preventive and Education section managers** (Gross salary and other allowances) *Allocation 25% operations* | 2,523 | 2*12*0.25 | 15,138 | 100% | 100% | 100% | 100% |
| **Senior administrative officer** (*Gross salary and other allowances)* *Allocation 100% operations* | 1,537 | 1*12 | 18,444 | 100% | 100% | 100% | 100% |
| **Administrative officers** (*Gross salary and other allowances)* Allocation 100% | 647 | 2*12 | 15,528 | 100% | 100% | 100% | 100% |
| **Chief accountant** (*Gross salary and other allowances)*  Allocation 100% | 2,268 | 1*12 | 27,216 | 100% | 100% | 100% | 100% |
| **Accountants** (*Gross salary and other allowances)* Allocation 100% | 647 | 2*12 | 15,528 | 100% | 100% | 100% | 100% |
| **Senior procurement officer** (*Gross salary and other allowances*) Allocation 100% | 1,174 | 1*12 | 14,088 | 100% | 100% | 100% | 100% |
| **Procurement officer** (*Gross salary and other allowances)* Allocation 100% | 647 | 1*12 | 7,764 | 100% | 100% | 100% | 100% |
| **Planning officer** (*Gross salary and other allowances*) A*llocation 100%* | 911 | 1*12 | 10,932 | 100% | 100% | 100% | 100% |
| **Lawyer**; *Allocation 30%* | 985 | 1*12*0.3 | 3,546 | 100% | 100% | 100% | 100% |
| **Legal officer**; *Allocation as lawyer* | 647 | 1*12*0.3 | 2,329 | 100% | 100% | 100% | 100% |
| **Senior IT computing officer** (*Gross salary and other allowances)* A*llocation 100%* | 911 | 1*12 | 10,932 | 100% | 100% | 100% | 100% |
| **IT computing officer** (*Gross salary and other allowances*) Allocation 100% | 647 | 2*12 | 15,528 | 100% | 100% | 100% | 100% |
| **Operations manager’s secretary** (*Gross salary and other allowances*). *Allocation as Operations manager* | 483 | 1*12 | 5,796 | 100% | 100% | 100% | 100% |
| **Director’s secretary;** *Allocation as director* | 483 | 1*12*0.4 | 2,318 | 100% | 100% | 100% | 100% |
| **Prevention and Education managers’ secretary;** *Allocation as corresponding managers* | 483 | 2*12*0.25 | 2,898 | 100% | 100% | 100% | 100% |
| **Receptionist** (*Gross salary and other allowances*) Allocation 100% | 483 | 1*12 | 5,796 | 100% | 100% | 100% | 100% |
| **Drivers** (*Gross salary and other allowances*) Allocation 100% | 306 | 2*12 | 7,344 | 100% | 100% | 100% | 100% |
| **Cleaners** (*Gross salary and other allowances*) Allocation 100% | 306 | 4*12 | 14,688 | 100% | 100% | 100% | 100% |
| **Human resources - Zonal offices** | Various | Various | 261,897 | 100% | 100% | 100% | 100% |
| **Law enforcement and inspection – Zonal level** | Various | Various | 90,370 | 100% | 100% | 100% | 100% |
| **F.2. Promotion and advocacy – Zonal offices** | Various | Various | 225,761 | 112,880 | 225,761 | 112,880 | 112,880 |
| **F.3. Program supplies** |  |  | 0 | 100% | 100% | 100% | 100% |
| **F.4. Rent, equipment and office supplies** | Various | Various | 79,560 | 100% | 100% | 100% | 100% |
| **F.5. Utilities** | Various | Various | 89,310 | 41,615 | 41,615 | 47,070 | 41,615 |
| **Sub-total F** |  | 1*0.20 | **197,967** | **165,852** | **188,428** | **166,943** | **165,852** |
| **TOTAL** |  |  | **443,635** | **415,712** | **423,294** | **416,803** | **415,712** |

**Table E: Intervention cost of tobacco tax increase**

|  | **Price (US$)** | **Quantity** | **Year 1** | **Year 2** | **Year 3** | **Year 4** | **Year 5** |
| --- | --- | --- | --- | --- | --- | --- | --- |
| 1. **Strategy development and evaluation** |  |  |  |  |  |  |  |
| **A.1. Law development** *based on standard cost norm to develop a law* | 122,683 | 1 | 122,683 | 0% | 0% | 0% | 0% |
| **A.2. Decree development** *(to guide the implementation of the law) (same basis as above)* | 22,852 | 1 | 22,852 | 0% | 0% | 0% | 0% |
| **Sub-total A** |  |  | **145,535** | **0** | **0** | **0** | **0** |
| 1. **Human resources** |  |  |  |  |  |  |  |
| **Steering committee** *(9 members, no salary, meetings allowance only (Tshs 200,000 per person), 2 meetings/year). Allocation: 10% on taxation policy* | 135 | 9*2*0.1 | 243 | 100% | 100% | 100% | 100% |
| **Commissioner General** (Gross salary and allowances) *allocation to LTD based on the number of personnel in the department* - (123/3831) *and to tobacco intervention based on percentage of tobacco to other revenue collection in the LTD* - 4.124% | 11,439 | 1*12*0.0012 | 165 | 100% | 100% | 100% | 100% |
| **Deputy Commissioner General** (Gross salary and allowances) *allocation as Commissioner General* | 8,075 | 1*12*0.0012 | 116 | 100% | 100% | 100% | 100% |
| **LTD commissioner,** *Gross salary and allowances, Allocation to tobacco based on revenue collection in the LTD - 4.124%* | 6,242 | 1*12*0.04 | 2,996 | 100% | 100% | 100% | 100% |
| **LTD deputy commissioner,** *Gross salary and allowances, Allocation as LTD commissioner* | 3,794 | 1*12*0.04 | 1,821 | 100% | 100% | 100% | 100% |
| **Tax officers,** *Gross salary and allowances, Allocation as LTD commissioner* | 2,195 | 100*12*0.04 | 105,360 | 100% | 100% | 100% | 100% |
| **Support staff,** *Gross salary and allowances, Allocation as LTD commissioner* | 694 | 20*12*0.04 | 6,662 | 100% | 100% | 100% | 100% |
| **B.3. Law enforcement and inspection** |  |  |  |  |  |  |  |
| 1 dedicated inspector at General Tax Bureau - 0.25FTE | 1,346 | 1*12*0.25 | 4,038 | 100% | 100% | 100% | 100% |
| Enhanced personnel at provincial tax bureau (0.1 FTE/district, 169 districts) | 673 | 169*12*0.1 | 136,484 | 100% | 100% | 100% | 100% |
| Tobacco smuggling control at 36 entry & border points (2 staff/spot, 0.05FTE) | 402 | 72*12*0.05 | 17,366 | 100% | 100% | 100% | 100% |
| Staff travelling allowance to supervision | Various | 0.04 | 17,672 | 100% | 100% | 100% | 100% |
| **Sub-total B** |  |  | **292,923** | **292,923** | **292,923** | **292,923** | **292,923** |
| 1. **Promotion, media and advocacy** |  |  |  |  |  |  |  |
| **Workshops, seminars, travelling expenses, advertising, publications etc.,** *allocation to LTD based on personnel (0.03) and to tobacco based on revenue collection (0.04)* | 761,782 | 1*0.0012 | 914 | 100% | 100% | 100% | 100% |
| **Training -** *allocation number of personnel from LTD who attended the trainings - 47/1045, and to tobacco according to revenue collections (0.04)* | 319,583 | 1*0.002 | 639 | 100% | 100% | 100% | 100% |
| **Sub-total C** |  |  | **1,553** | **1,553** | **1,553** | **1,553** | **1,553** |
| 1. **Program supplies** |  |  | 0 | 0 | 0 | 0 | 0 |
| **Sub-total D** |  |  | **0** | **0** | **0** | **0** | **0** |
| 1. **Office rent, equipment and supplies,** *Allocation to LTD based on personnel and to tobacco interventions based on proportion of revenue collection* | Various | Various | 4,372 | 4,372 | 4,372 | 4,372 | 4,372 |
| **Sub-total E** |  |  | **4,372** | **4,372** | **4,372** | **4,372** | **4,372** |
| 1. **Administration** *Allocation to LTD based on personnel and to tobacco interventions based on proportion of revenue collection* |  |  |  |  |  |  |  |
| **F.1. Human resources** | Various | Various | 3,367 | 100% | 100% | 100% | 100% |
| **F.2. Utilities** | 1,722,204 | 1*0.0012 | 2,067 | 100% | 100% | 100% | 100% |
| **Sub-total F** |  |  | **5,434** | **5,434** | **5,434** | **5,434** | **5,434** |
| **TOTAL** |  |  | **449,817** | **304,282** | **304,282** | **304,282** | **304,282** |
